# Soft Tissue Sarcoma: An Insight on Biomarkers at Molecular, Metabolic and Cellular Level

**DOI:** 10.3390/cancers13123044

**Published:** 2021-06-18

**Authors:** Serena Pillozzi, Andrea Bernini, Ilaria Palchetti, Olivia Crociani, Lorenzo Antonuzzo, Domenico Campanacci, Guido Scoccianti

**Affiliations:** 1 Medical Oncology Unit, Careggi University Hospital, Largo Brambilla 3, 50134 Florence, Italy; lorenzo.antonuzzo@unifi.it; 2 Department of Biotechnology, Chemistry and Pharmacy, University of Siena, Via Aldo Moro 2, 53100 Siena, Italy; andrea.bernini@unisi.it; 3 Department of Chemistry Ugo Schiff, University of Florence, Via della Lastruccia 3, 50019 Sesto Fiorentino, Italy; ilaria.palchetti@unifi.it; 4 Department of Experimental and Clinical Medicine, University of Florence, Largo Brambilla 3, 50134 Florence, Italy; olivia.crociani@unifi.it; 5 Department of Health Science, University of Florence, Largo Brambilla 3, 50134 Florence, Italy; domenicoandrea.campanacci@unifi.it; 6 Department of Orthopaedic Oncology and Reconstructive Surgery, University of Florence, Careggi University Hospital, Largo Brambilla 3, 50134 Florence, Italy; scocciantig@aou-careggi.toscana.it

**Keywords:** soft tissue sarcoma, prognostic/predictive biomarker, oncogene, immune checkpoints, lncRNA, metabolite

## Abstract

**Simple Summary:**

Soft tissue sarcoma is a rare mesenchymal malignancy. Despite the advancements in the fields of radiology, pathology and surgery, these tumors often recur locally and/or with metastatic disease. STS is considered to be a diagnostic challenge due to the large variety of histological subtypes with clinical and histopathological characteristics which are not always distinct. One of the important clinical problems is a lack of useful biomarkers. Therefore, the discovery of biomarkers that can be used to detect tumors or predict tumor response to chemotherapy or radiotherapy could help clinicians provide more effective clinical management.

**Abstract:**

Soft tissue sarcomas (STSs) are a heterogeneous group of rare tumors. Although constituting only 1% of all human malignancies, STSs represent the second most common type of solid tumors in children and adolescents and comprise an important group of secondary malignancies. Over 100 histologic subtypes have been characterized to date (occurring predominantly in the trunk, extremity, and retroperitoneum), and many more are being discovered due to molecular profiling. STS mortality remains high, despite adjuvant chemotherapy. New prognostic stratification markers are needed to help identify patients at risk of recurrence and possibly apply more intensive or novel treatments. Recent scientific advancements have enabled a more precise molecular characterization of sarcoma subtypes and revealed novel therapeutic targets and prognostic/predictive biomarkers. This review aims at providing a comprehensive overview of the most relevant cellular, molecular and metabolic biomarkers for STS, and highlight advances in STS-related biomarker research.

## 1. Introduction

Soft tissue sarcomas (STSs) are rare neoplasms, accounting for fewer than 1% of all neoplasms [1]. More than 80 malignant or intermediate (rarely metastasizing) histotypes are currently recognized [1]. In recent years, new histological entities or revisions of previous subclassifications have been introduced due to the advances in genetics and molecular diagnostics [2], and following the continuously growing achievements in these fields, further modifications in the classification and diagnostic approach to sarcomas are to be expected in the future.

An incidence rate of STS ranging from 4 to 6 cases per 100,000 people per year was reported for European population from an analysis of tumor national registries [3]; a similar average rate (5 cases per 100,000 per year) is reported by the World Health Organization [1]. STSs can affect any body site, with extremities accounting for 75% and the trunk wall and retroperitoneum accounting for 10% of diagnosed tumors [1]. STSs can arise in any age, with an increasing rate in older patients [1,4,5]. Differences in age of presentation are an established finding, with some histotypes almost limited to childhood, such as embryonal rabdomyosarcoma, and others are definitely more frequent in old age, such as myxofibrosarcoma. No clear sex prevalence has been reported, with some authors reporting a slightly higher rate in males [4] and other in females [3,5].

As in most tumors, particularly in rare tumors, the etiology of STS is still largely unknown. There is, however, historical evidence of the association between sarcomas and various genetic syndromes as well as with radiotherapy; moreover, there are data indicating a possible role of environmental factors predisposing sarcoma development. Recognized predisposing genetic diseases associated with STSs are Li-Fraumeni syndrome (rhabdomyosarcoma), retinoblastoma (different histotypes, with leiomyosarcoma as the most frequent [6]), and neurofibromatosis (malignant peripheral nerve sheath tumors).

Radiation treatment can lead to a late occurrence of STSs; 3 to 5% of STSs can be considered radiation-induced [1,7]. Other historical exposures to radiation have been linked to the occurrence of sarcoma, such as workers dealing with radium in watch factories in the 1920’s.

Viral infection associated with immunodepression was shown to predispose to sarcomagenesis in HIV patients not only for Kaposi’s sarcoma, but also for leiomyosarcoma after infection by Epstein–Barr virus [8].

Additionally, exposure to some chemicals has been claimed as a predisposing factor for sarcoma onset, even if data reported are not univocal. Particularly, dioxins from waste incinerators have been reported as a possible predisposing factor [9,10], as well as phenoxy herbicides and other pesticides used in agriculture, but a meta-analysis of the literature could neither confirm nor rule out a possible role of these substances in sarcomagenesis [11].

Surgical excision with wide margins remains the mainstay of treatment for STSs. Surgery must be performed in specialized centers with specific expertise in sarcoma surgery, and referral to these centers is highly recommended for any STS and also for any soft tissue mass suspected to be a sarcoma (i.e., a superficial mass > 5 cm or a deep mass of any size [12]) because the diagnostic biopsy is the first step of treatment and can heavily influence subsequent surgery if inadequately performed. Several studies have confirmed that patients primarily treated in sarcoma centers show better results than patients initially treated elsewhere [5,13]. Specific expertise is fundamental not only for surgery, but for any step of sarcoma treatment, from histopathological diagnosis to any component of the multimodality treatment and follow-up. In addition to surgery, a consistent role is universally recognized for the use of radiotherapy, but it is still debated whether it is more efficient to deliver radiant treatment before or after surgery and which parameters should identify the tumors more likely to benefit from radiotherapy. The role of chemotherapy is more controversial, with a better control of disease reported by some authors both in a neoadjuvant or adjuvant settings of anthracycline-based regimens, along with other studies not able to confirm this finding (see meta-analyses in references [14] and in [15]). In the last decade, substantial efforts have been made to identify histotype-targeted therapies with promising preliminary results, which hopefully will be improved in the near future. Specific activity of medical treatment in selected histotypes has been proposed for trabectedin in mixoid or round cell liposarcoma and leiomyosarcoma, gemcitabine and taxanes for angiosarcoma, gemcitabine and docetaxel for undifferentiated pleomorphic sarcoma (UPS), and leiomyosarcoma and ifosfamide for synovial sarcoma. Tyrosine kinase inhibitors were recently introduced for the treatment of advanced STSs not responding to more traditional medical treatment, with interesting results, as well as immunotherapy treatments such as immune checkpoint inhibitors (ICIs), vaccination against tumor-related antigens or dead cells, and engineered T cells. A recent, accurate summary of ongoing experiences in all these fields can be found in [1,16].

Due to the heterogeneity and complexity of STSs and their response to treatment, a multidisciplinary approach to any single case is mandatory to define a tailored therapeutic plan with a case-specific evaluation, in order to decide which treatments must be applied and in which order.

Reliable biomarkers to enable the screening and surveillance of STSs are still unavailable. Ongoing molecular characterization of sarcoma pathogenesis is of utmost importance to shed light on etiology and diagnosis issues and to try to find new and more efficient therapeutical approaches. The aim of this review is mainly to highlight the state of the art and the more recent achievements in the understanding of the most relevant metabolic, genetic and molecular biomarkers related to STS; these will be detailed in the following sections, bearing in mind their clinical relevance.

## 2. Molecular Markers Associated with Prognosis

Sarcomas have not been the subject of larger systematic studies on how tumor behavior relates to characteristics of the tumor microenvironment. There is consequently an urgent need for identifying suitable molecular targets, not only in tumor cells but also in the tumor microenvironment.

### 2.1. Microenvironment

#### 2.1.1. Markers of Infiltration

##### B Cells

B cells play a fundamental role in tumor microenvironments by producing antibodies that mediate antibody-dependent cell cytotoxicity and phagocytosis against neoplastic cells [17]. A study that evaluated the presence of B cells in 249 STSs showed that infiltration is associated with a better prognosis [18], and a concordant result emerged from an analysis carried out evaluating CD20^+^ in 33 patients with STS and confirmed by the evaluation of the expression of the MS4A1 gene (which codes for CD20^+^) in 265 patients [19]. Numerous studies have registered a low frequency of CD20^+^ cells infiltrating STSs [19,20,21], and in one study on rhabdomyosarcoma, CD20^+^ tumor-infiltrating cells were identified only at the periphery of the tumor [22]. Recently, a study conducted on 608 STSs found that the 18% of sarcomas with a high presence of B cells were associated with better survival and response [23].

##### T Cells

**Figure 1:**
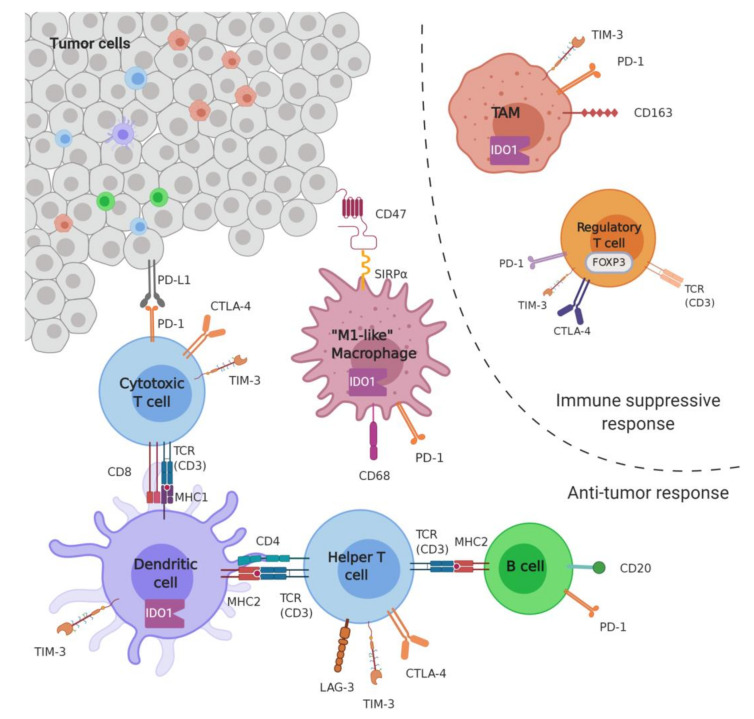
Schematic illustrating the different markers of infiltration and the immune checkpoints reported for different malignancies including STSs. Reprinted with permission from reference [27]. TAM: tumor-associated macrophage. Immune cells in the tumor microenvironment might have immune-suppressive activity or exhibit anti-tumor responses. CD8^+^ cytotoxic T cells are activated via CD8/ MHC I/ T cell receptor (CD3 or TCR) complexes and helper T cells through the CD4/ MHC II/ TCR complex by dendritic cells. Helper T cells activate CD20^+^ B cells, inducing plasma cell differentiation and antibody class switching. Macrophages can be pro-inflammatory and anti-tumor CD68^+^ (M1), or anti-inflammatory and pro-tumor CD163^+^ (M2) macrophages. FOXP3^+^ regulatory T cells are immunosuppressive. Immune checkpoints such as PD-1, CTLA-4, TIM-3, and LAG-3 can be found on the surface of a variety of immune cells. The PD-1/PD-L1 axis and CD47/SIRPα axis are two immune checkpoint pathways that interact with tumor cells.

Tumor-infiltrating T cells, known also as tumor-infiltrating lymphocyte (TILs), play a key role in the immune response against cancer cells. The presence of CD8^+^ and CD4^+^ T cells is associated with an improvement in survival and response to therapies [24], whereas the presence of FOXP3^+^ T cells is associated with an immunosuppressive response [25] (Figure 1). The expression of mRNA in order to quantify TILs was evaluated both on The Cancer Genome Atla (TGCA) dataset and on an independent STS cohort, highlighting a positive correlation between mRNA levels and global survival [26]. Many studies have found a greater number of infiltrating CD8^+^ than FOXP3^+^, and infiltration is greater in copy number-driven subtypes than those associated with translocation [27]. The CD4^+^ cell number also correlates with longer survival in patients with large resection margins [28]. Newly diagnosed STSs have higher CD8^+^ counts, and decreasing CD8^+^ counts at relapse are associated with poor survival [18]. Regarding synovial sarcoma, there are conflicting data. In one study, CD8^+^ infiltration was described in 45% [29]; in another, in 97% of cases, a high infiltration of CD8^+^ cells was associated with a longer survival [30]. In Ewing’s sarcoma and angiosarcoma, high levels of CD8^+^ cells are associated with increased survival [31,32]. A high number of CD8^+^ has also been detected in myxofibrosarcoma and pleomorphic sarcoma [33].

##### Macrophages

STSs are more often infiltrated by macrophages than by lymphocytes [3]. Tumor-infiltrating macrophages (TIMs) (CD163^+^ (M2) and CD68^+^ (M1)) play a key role in the tumor microenvironment [19]. A study carried out on 1242 sarcomas showed that the subtypes with the greatest presence of TIMs are leiomyosarcoma, myxofibrosarcoma, dedifferentiated liposarcoma and undifferentiated pleomorphic sarcoma; moreover, a higher level of macrophages was observed in the copy number driven subtypes [19]. The most common phenotype is M2, and low CD163^+^ levels are associated with favorable survival in synovial sarcoma [15]; in embryonic rhabdomyosarcoma, on the other hand, high levels of CD163^+^ are positively associated with survival [22]. Alterations in CD68^+^, CD163^+^ density and CD163^+^:CD68^+^ ratio have been observed in patients responding to neoadjuvant chemotherapy [34].

#### 2.1.2. Immune Checkpoints

The immune checkpoints that have an inhibitory effect on the immune system and play a role in STSs include the receptor PD-1 and its ligand PDL1, the enzyme 2,3-dioxygenase (IDO), the T cell immunoglobulin and mucin domain 3 receptor TIM3 and its ligand galectin-9 (Gal9), the lymphocyte activation gene-3 receptor (LAG3) and its ligand MHC II, and the receptor signal regulatory protein α (SIRPα) and its ligand CD47.

##### PD1-PDL1

The programmed cell death-1 (PD1) checkpoint is a membrane receptor expressed by T lymphocytes and B lymphocyte precursors (Figure 1). Its two ligands are PDL1 and PDL2, where PDL1 can be expressed by cancerous cells and the PD1–PDL1 bond promotes tumor evasion from the immune system by inducing antigen-specific T cell apoptosis and inhibiting the apoptosis of regulatory T lymphocytes [35]. The PDL1 presence is relatively low in STS compared to other malignancies, and a number of studies have been carried out on the presence and role of this immune checkpoint in STSs, some of which have given contrasting results. One study on 234 patients highlighted how PDL1^+^ cell levels in synovial sarcomas are 15.7% [36]. Another study identified one case out of 62 PDL1^+^ liposarcomas and three cases out of 96 of rhabdomyosarcomas [37]. A study carried out on 48 cases of patients suffering from sarcomas including various subtypes of STSs, highlighting neoplastic PDL1^+^ cells in 20.8% of the analyzed cases [26]. A different study reports a percentage of PDL1^+^ STSs of 58%, and reports that the presence of PDL1 has no prognostic value from the point of view of overall survival [38], in contrast with data reported by another group who highlighted how PDL1^+^ patients have a worse prognosis compared to PDL1^+^ patients [39]. An increase in PDL1 levels has also been shown following pre-surgical radiotherapy [40]. Finally, with regard to the presence of infiltrating cells in PD1^+^ tumors, a study carried out on 105 samples demonstrated their presence in 65% of the cases tested [38].

##### Others (IDO, LAG3, TIM3)

IDO (Indoleamine 2,3-Dioxygenase 1): IDO is an enzyme involved in the catabolism of tryptophan (kynurenine pathway). IDO and kynurenine can be secreted by tumor cells into the microenvironment where they exert an immunosuppressive action by blocking M2 phenotype myeloid cells, suppressing the function of effector T lymphocytes, and stimulating the expansion and activation of regulatory T lymphocytes. Furthermore, their secretion leads to a depletion of tryptophan in the tumor microenvironment, favoring neoplastic growth [41]. In a study on the role of IDO1 in sarcomas, its expression was highlighted in 39.1% of STSs, and in 65.3% if we consider the subgroup with high levels of CD8^+^ TILs. Furthermore, the IDO1/KP signaling pathway contributes to an immunosuppressive phenotype of STSs and is involved in primary resistance to PD1 inhibitors [42].

TIM3–GAL9 (T cell immunoglobulin and mucin domain-containing protein 3–galectin9): TIM3 is an inhibitory T cell receptor and can be found in CD4^+^ and CD8^+^ T lymphocytes. Its Gal9 ligand can be expressed by tumor cells, and TIM3–GAL9 binding has inhibitory effects on T cells and the immune microenvironment [43]. In a study on immune checkpoints linked to CD8^+^ TILs, an elevated presence of TIM3^+^ cells was observed in myxofibrosarcoma and pleomorphic sarcoma compared to liposarcoma. Co-expression with PD1 is frequent [33].

LAG3 MHCII (lymphocyte activation gene-3–major histocompatibility complex class II): LAG3 can be expressed by T CD4^+^ and CD8^+^ lymphocytes, plasmocytoid dendritic cells, and natural killer cells. Its main ligand is MHCII, expressed either by antigen-presenting cells (APCs) or directly by cancerous cells. The LAG3–MHCII bond has an inhibitory effect on the immunologic components of the tumor microenvironment. In some cases, LAG3 can be found in a soluble form (sLAG3) [44]. In a study carried out on 163 STS-affected patients, it was found that the frequency of CD8^+^ and CD4^+^ T lymphocytes expressing LAG3 is higher than in healthy controls; moreover, the expression is correlated with the presence of TILs CD8^+^, with a severe prognosis and advanced tumor stage [45]. LAG3 is often co-expressed with PD1, and rarely with TIM3 [33].

SIRPα (signal regulatory protein α)–CD47: The SIRPα receptor is expressed by tumor-associated macrophages, and binding with the CD47 ligand expressed by tumor cells induces the phosphorylation of SIRPα with a final inhibitory effect on the phagocytosis process [46]. It has been reported that 82% of undifferentiated pleomorphic sarcoma, 78% of leiomyosarcoma, and 70% of Ewing’s sarcoma are negative for CD47, whereas high levels of CD47 were found in angiosarcoma (100% of the cells in 75% of the samples). In dedifferentiated liposarcoma, pleomorphic liposarcoma and epithelioid sarcoma, more than 90% of the tumor cells within the sample express CD47 in 71%, 64% and 63% of the samples, respectively. SIRPα+ macrophages were identified in 31.3% of total cases; the subtypes of STS most infiltrated by SIRPα+ macrophages are dedifferentiated liposarcoma (77% of cases), angiosarcoma (75%), and well-differentiated liposarcoma (65%), whereas the subtypes less infiltrated by SIRPα+ macrophages are fibromyxoid sarcoma (0% of cases), epithelioid sarcoma (13%), and synovial sarcoma (14%). Finally, in some cases, the expression of SIRPα was observed in sarcoma cells [47].

### 2.2. Genetics

#### 2.2.1. Tumor Mutation Burden

Tumor mutation burden (TMB) is defined as the number of somatic mutations per megabase of the genomic sequence analyzed. It can be divided into low TMB (1–5 mutations/Mb), intermediate (6–19 mutations/Mb) and high (≥20 mutations/Mb). Together with the instability of microsatellites, it is often considered a biomarker positively correlated with the response to immunotherapy [48]. Numerous studies show that in sarcoma, and also specifically in STS, the TMB tends to be low. A study carried out on 206 cases of STS through whole exome sequencing (WES) showed an average TMB equal to 1.06 mutations/Mb [49]. A further study in 68 STS patients reported a relatively low TMB, with a median of 2.05 mutations/Mb and a range of 0 to 15.5 mutations/Mb. By dividing the patients into two groups based on the median value of TMB, no significant differences were observed from the point of view of clinical–pathological characteristics; instead, a significant difference (*p* = 0.015) was observed in overall survival [50]. Regarding synovial sarcoma, a study on 208 cases reported a median value of TMB of 1.7 mutations/Mb, with only 1% of cases having high TMB (≥20) [51]. There are also data on studies with small numbers of patients with synovial sarcoma: one with 16 patients reported a median TMB of 1.7 mutations/Mb with only one patient with high TMB [52]; another study with 7 patients reported a mean value of TMB equal to 8.1 ± 4.4 [53]. In Ewing’s sarcoma, one study demonstrated TMB values of fewer than two mutations/Mb, with a 2–3-fold increase in relapsing cases [54,55]. Low values of TMB have also been found in alveolar STS [56].

#### 2.2.2. Microsatellite Instability

Microsatellite instability (MSI) is a condition in which there are altered numbers of repetitions of short DNA [57]. The presence of MSI in some types of cancer appears to be related to specific clinical–pathological features, such as localization, tumor lymphocyte infiltration, differentiation, frequency of distant metastases, and prognosis [58].

In 1994, the first data on MSI in STS highlighted their presence in 11% of tested cases [55]. Subsequently, a study evaluated the absence of MSI in eight cases of low-grade STS and the presence of MSI in eight high-grade cases [59], and another survey carried out on 40 samples highlighted the presence of MSI in 25% of cases [60]. A recent study carried out on 71 patients evaluated the presence of MSI utilizing five markers, and only three cases of STS presented MSI, showing instability in only one of the tested markers (4.2%) [61]. The low percentages and the non-concordant data between the various studies indicate that the instability of microsatellites in STS does not play a primary role.

#### 2.2.3. Molecular Biomarkers

##### Tumor Suppressor Genes

There is a lot of evidence that shows a correlation between genetic events that lead to the loss of function of oncosuppressor genes and the incidence and evolution of STSs. The most involved and characterized oncosuppressors are TP53, RB1, NF1, PTEN, CDKN2A, SMARCB1, and ATRX.

TP53 (tumor protein 53): The TP53 gene encodes for protein p53, a transcription factor that, once stabilized and active, can act on target genes involved in cell cycle arrest, in apoptotic processes, and metabolism [62]. The loss of its function is associated with many neoplasms and can occur through a number of processes: loss of function mutations, deletions, and DNA binding domain missense mutations [63]. From a phenotypic point of view, p53 can also be inactivated by the overexpression of MDM2 [64]. Amongst the STSs that present alterations of TP53 are leiomyosarcomas, liposarcomas, undifferentiated pleomorphic sarcomas, synovial sarcomas, rhabdomyosarcomas and angiosarcomas [65,66].

Li Fraumeni syndrome, characterized by a germinal mutation of TP53, is considered a predisposing factor for many malignancies, and it has been demonstrated that the incidence of rhabdomyosarcomas and liposarcomas in patients positive for this genetic syndrome is significantly greater with respect to their incidence in the general population [66].

RB1 (RB transcriptional corepressor 1): The RB1 gene is an oncosuppressor that encodes for the Rb protein, which physiologically regulates the cell cycle blocking S phase entry. Loss of function can occur following direct RB1 gene mutations or mutations that cause an increase in phosphorylated Rb protein levels [67]. Amongst STSs most frequently associated with the loss of function of the RB1 gene are the leiosarcoma, the undifferentiated pleomorphic sarcoma, and the myxofibrosarcoma [65]. Retinoblastoma, a genetic syndrome caused by a germinal mutation of the RB1 gene, is considered a predisposing factor for many malignancies such as STSs, which are usually diagnosed with a delay of about 10–50 years with respect to the diagnosis of the syndrome itself. The strongest association between the syndrome and STS has been highlighted in leiomyosarcomas [68].

NF1 (neurofibromin 1): The NF1 gene is considered to be an oncosuppressor gene that encodes for neurofibromin protein 1, a negative feedback regulator of the RAS–MAPK signaling pathway. Mutations and small deletions on this gene are responsible for type 1 neurofibromatosis [69]. Rhabdomyosarcoma prevalence in children with type 1 neurofibromatosis is twenty times higher compared to the general population; moreover, NF1-associated rhabdomyosarcomas often develop in the bladder and prostate [70]. The annual incidence of malignant tumors affecting the peripheral nerve sheaths in type 1 neurofibromatosis-affected patients is 0.16%, significantly higher when compared to that of the general population (0.001%) [70]. There is also evidence of a loss of function mutations of the NF1 gene in undifferentiated pleomorphic sarcoma and liposarcoma [65].

PTEN (phosphate and tensin homologous gene): The PTEN gene is considered an oncosuppressor gene whose principal function can be found in dephosphorylating PIP3 in the PIK3/PTEN/Akt/mTOR signaling pathway [71]. A study carried out on bone and soft tissue malignancies demonstrated how the loss of function of PTEN is present in 38.6% of cases, and the most involved STSs are leiomyosarcoma, rhabdomyosarcoma and epithelial sarcoma [72]. Another study highlighted that mutations and deletions on the PTEN gene can be found in 2–10% of STSs [73].

CDKN2A (cyclin-dependent kinase inhibitor 2A): The CDKN2A gene is considered to be an oncosuppressor gene, and encodes for two genic products post alternative splicing: p16 (ink4a), a negative regulator of CDK4 and CDK6; and p19 (Arf), a negative regulator of MDM2 [74]. Loss of function mutations and deletions at this level have been identified in peripheral nerve sheath malignancies, with a higher incidence in cases associated with type 1 neurofibromatosis [75].

SMARCB1 (SWI/SNF-related, matrix-associated, actin-dependent regulator of chromatin, subfamily B, member 1): The SMARCB1 gene is considered to be an oncosuppressor gene, and encodes for the homologous protein, part of the SWI/SNF remodeling complex protein. Such a gene is often inactivated in epithelial sarcomas [76].

##### Oncogenes

In STSs, the presence of oncogenes is less relevant compared to that of oncosuppressor genes. A genomic analysis carried out in order to characterize STSs highlighted that somatic copy number alterations (SCNAs) often involve MDM2–P53 and P16–CDK4–RB1 signaling pathways; with regard to oncogenes, MDM2 is, by definition, amplified in 100% of dedifferentiated liposarcomas, and can also be found in other STS subtypes. CDK4 is commonly amplified, with a higher incidence (86%) in dedifferentiated liposarcoma [16]. In dedifferentiated liposarcoma, other amplifications have also been documented at HMGA2 (76% of tested cases), FRS2 (96%), NAV3 (60%) and at the gene level that inhibit adipocyte differentiation (JUN, DDIT3, PTPRQ, YAP1, CEBPA) [49]. An elevated PI3K–AKT–mTOR activation and amplification of MYOCD has been observed in leiomyosarcoma [49]. In the pleomorphic undifferentiated sarcoma and in myxofibrosarcoma, amplifications of CCNE1 (10%), VGLL3 (11%), and YAP1 (3%) have been observed [49]. In rhabdomyosarcomas, activating mutations have been observed at the PI3K–AKT–mTOR and RAS–RAF–MAPK signaling pathway [77,78]. Moreover, mutations of the FGFR4 gene have been highlighted in 17% of studied cases [79]. In synovial sarcoma, gene expression analysis revealed high levels of EGFR, SSX [80], ERBB2, IGFBP2, and IGF2 expression [81]: this subtype of sarcoma is characterized by a translocation (X,18; p11,q11) which determines a fusion gene between SS18 and SSX1 (or SSX2) that exerts a key role in this neoplasm [82].

Furthermore, a cytogenic analysis of STSs identified chromosomal translocations that induced the encoding of specific tumor subtype oncoproteins: Ewing’s sarcoma (EWS–FLI-1 fusion), clear cell sarcoma (EWS–ATF1 fusion), myxoid sarcoma (TLS–CHOP fusion), alveolar rhabdomyosarcoma (PAX3–FHKR fusion), and small round cell desmoplastic tumor (EWS–WT1 fusion) [83,84,85,86,87]. In a study carried out with next-generation sequencing on 25 cases of STS, Myc amplification was observed in 33% of the cases, MAP2K4 in 20%, and CNV and FGFR amplification in 40% of the studied cases. In this same study, with an untargeted analysis, SNVs were found at the FLT4, NOTCH1, IGF1R, and PIK3R1 level [88].

##### MicroRNA

Several studies have highlighted identifiable microRNA (miRNA) in various STS subtypes, and have hypothesized their possible role as detectable biomarkers in tissue and liquid biopsy (LB).

Liposarcoma: In dedifferentiated liposarcoma (DDLS), miR-21, miR-26, miR-218-1, and miR-144 microRNA are upregulated, whereas miR-143, miR-145, miR-1238 are downregulated when compared to healthy adipose tissue [89,90]. miR-193b is downregulated compared to healthy adipose tissue and well-differentiated liposarcoma (WDLS) [91]. Furthermore, miR-3613-3p levels are significantly higher in blood samples of patients suffering from dedifferentiated liposarcoma [92]. In both the well-differentiated and dedifferentiated liposarcoma, miR-155 is highly expressed [93]. The myxoid liposarcoma (MLS) is characterized by high levels of expression of miR-9, miR-891a and miR-888, and low levels of miR-486 [94]. In the pleomorphic liposarcoma (PLS) miR-1249, miR-296-5p and miR-455-5p are upregulated, whereas miR-200b, miR-200 and miR-139-3p are downregulated with respect to the control tissue [90]. miR26a-2c miRNA levels are amplified in well-differentiated liposarcoma, dedifferentiated and myxoid, and such amplification is correlated to poor survival [95].

Rhabdomyosarcoma: Given the origin of such sarcoma, numerous miRNAs involved in physiologic muscular differentiation are altered: miR-28a and miR-203 are downregulated in all forms of rhabdomyosarcoma [96]; in pleomorphic rhabdomyosarcoma, miR-1 and miR-133 are downregulated; alveolar rhabdomyosarcoma is characterized by high levels of miR-335 [97]; finally, with regard to muscle-specific miRNA, low expression levels of miR-206 have been highlighted in alveolar and embryonic rhabdomyosarcoma [98]. With regard to non-muscle-specific miRNA, high levels of miR-9 and low levels of miR-200c have been highlighted in alveolar rhabdomyosarcoma [99], whereas miR-29 is downregulated in all forms of rhabdomyosarcoma [100]; high expression levels of the miR-17-92 cluster are correlated with a poor prognosis; and finally, the signaling pathway tied to miR-485-3p-Top2α-NF-YB is involved in mechanisms of therapy sensitivity [101,102]. Furthermore, several miRNAs (miR-1, miR-133a, miR-133b and miR-206) have been identified as circulating biomarkers in sera of patients suffering from rhabdomyosarcoma [103].

Leiomyosarcoma: microRNAs miR-1 and miR-133a/b play a key role in the myogenesis and proliferation of myoblasts, and are significantly overexpressed, whereas miR-206 is downregulated [104]. Moreover, miR-221 is upregulated in uterine leiomyosarcoma [105]. A group of miRNAs (miR-199b-5p, miR-320a, miR-199a-3p, miR-126, and miR-22) is conversely detectable in patients’ sera [106].

Synovial sarcoma: Low expression levels of miR-143 have been highlighted, associated with the production of oncoprotein SS18-SSX1 [97]. miR-183 is upregulated, stimulating the miR-183–EGR1–PTEN signaling pathway [107].

Sera levels of miR-92b-3p are correlated with tumor burden. A panel of miRNA (miR-99a-5p, miR-146b-5p, miR-148b-3p, miR-195-5p, miR-223-3p, miR-500b-3p and miR-505-3p) is identifiable in peripheral blood samples of patients suffering from synovial sarcoma, and can be utilized as a diagnostic biomarker both for differentiating it from other sarcoma subtypes and for predicting metastatic events [108].

Malignant neoplasm of peripheral nerve sheaths: High levels of expression of miR-10b, miR-210, miR-339-5p and miR-199a/214 cluster [109,110], and low levels of miR-34a and miR-204 [104,111] have been identified. miR-30d is downregulated, and this is tied to the EZH2–miR-30d–KPNB1 pathway [112].

Angiosarcoma: miR-515-5p, miR-517a, miR-518b, miR-519a, miR-522 and the miR-17-92 cluster miRNA are upregulated in angiosarcoma. Moreover, miRNAs of chromosome 19, usually expressed at high levels in the placenta, are upregulated [107,113].

Fibrosarcoma: Studies related to microRNA in fibrosarcoma have been carried out using the human cell line HT-1080, where the miR-29 family activates MMP-2 which plays a role in tumor suppression; miR-520c and miR-373 activate the Ras/Raf/MEK/Erk and NF-κB signaling pathway, promoting the migration and invasion of cancer cells [114,115]. Undifferentiated pleomorphic sarcoma: miR-126, miR-223, miR-451 and miR-1274b are significantly upregulated, and miR-100, miR-886-3p, miR-1260, miR-1274a, and miR-1274b significantly downregulated when compared with control mesenchymal stem cell lines. Furthermore, miR-199-5p and miR-320a can be used to differentiate between undifferentiated pleomorphic sarcoma and leiomyosarcoma [106]. Finally, a negative correlation was observed between miR-138 overexpression and metastasis-free survival [116].

Epithelial sarcoma: In this type of STS, a panel of miRNAs (miR-206, miR-381, miR-671-5p and miR-765) has been identified to be overexpressed, and three of these (miR-206, miR-381, and miR-671-5p) induce the silencing of SMARCB1 mRNA [117].

Kaposi sarcoma: Some tumor-suppressor miRNAs, such as miR-155, miR-220/221, the let-7 family and the miR-221/222 cluster, are downregulated in this form of sarcoma, whereas miR-31 is found to be upregulated [118,119]. A specific feature of Kaposi’s sarcoma is the high expression of the pre-miRNA related to miR-24-2 and its use as a biomarker is being evaluated [120].

Other STSs: STSs share the correlation between the expression of miR-210 and a severe prognosis; this correlation is more significant in female patients [121].

##### Long Non-Coding RNA

There is little evidence regarding the role of long non-coding RNA (lncRNA) in STSs and their possible use as molecular biomarkers.

Upregulation of HOTAIR lncRNA has been observed in chondrosarcoma and chondrosarcoma cell lines (including soft-tissue-derived forms such as mesenchymal and myxoid chondrosarcoma), correlated with tumor stage and with a severe prognosis. Its downregulation, on the other hand, is related to an inhibition of cell growth through the arrest in the G0/G1 phase of the cell cycle and the induction of apoptosis. HOTAIR induces DNA methylation of miR-454-3p by recruiting enhancer EZH2 and DNA methyltransferase 1 in the promoter regions of miR-454-3p by silencing their expression [122].

In retroperitoneal liposarcoma, PIRLS lncRNA is overexpressed and carries out its action by binding to the T cell leukemia 1A (TCL1A) protein, thus suppressing the P53-mediated signaling pathways and activating the expression of MDM2 and AKT [123]. A large-scale genomic analysis, based on RNAseq expression data and conducted on a large cohort of patients with STS, selected and validated 10 lncRNAs with prognostic value, four of which were related to a favorable prognosis (LINC00680, AC006129.2, RP11-274B21.9 and RP11-713P17.3) and six to a severe prognosis (RP11-560J1.2, AP001432.14, RP4-665J23.1, RP11-230G5.2, BACH1-IT2, and RP11504A18.1). The predictive score of the lncRNA panel is able to predict the survival of patients with STS regardless of the clinicopathological characteristics, giving them a possible role as a molecular biomarker [124].

##### Telomere-Maintenance Mechanism

The maintenance of telomere length is commonly achieved through activation of the TERT telomerase gene, and this leads to replicative immortality [125].

The two most common activating mutations occur at the telomerase promoter level and are two cytosine thymine transitions (C228T; C250T), common mutations in myxoid liposarcoma and are present in 11% of STSs [126,127].

Hypermethylation states were also observed at the promoter level of the TERT gene, with positive consequences on the maintenance of telomeres [128].

Finally, genome-wide sequencing experiments have shown rearrangements in the vicinity of TERT that lead to an increase in its expression, probably related to the capture of zone enhancers [129]. There is also an independent telomerase mechanism to maintain telomere length called the alternative lengthening of telomeres (ALT) mechanism [130]. The presence of this mechanism varies in the histological subtypes of STS: it is present in 63% of undifferentiated pleomorphic sarcoma, in 53% of leiomyosarcoma, in 33% of epithelioid sarcoma, in 24–26% of all liposarcomas [131,132] (0% well differentiated, 30% dedifferentiated, 5% myxoid and 80% pleomorphic) [70], in 14% of fibrosarcoma, and in 11–28% of angiosarcoma [133,134]. Furthermore, in liposarcoma and leiomyosarcoma, the presence of the ALT mechanism is associated with genomic instability, aggressive histological features, and a severe prognosis [134,135]. The ALT mechanism is significantly associated with the presence of inactivating mutations in the ATRX and DAXX genes, mutations present in 31% of undifferentiated STSs [49,136].

The main molecular markers in STSs that have been reported to be associated with the prognosis and mechanism of resistance to therapies are summarized in Table 1A,B.

## 3. Metabolite Biomarkers

**Figure 2:**
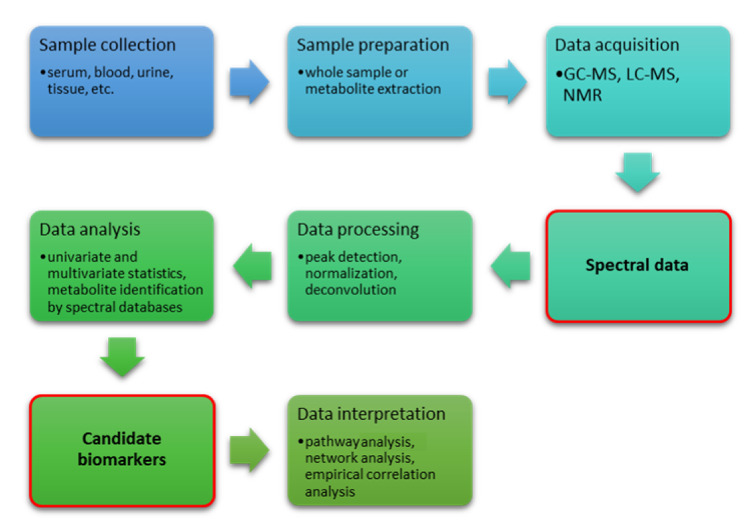
The workflow of metabolomics in sarcoma: both biofluids and tissue extract can be used as a whole or after metabolite extraction to reduce matrix complexity (steps 1–2); spectral data are first collected in a non-destructive way with samples by nuclear magnetic resonance followed by mass spectrometry, coupled either to gas chromatography or liquid chromatography (steps 3–4); both data from NMR and MS need extensive processing to resolve spectral overlapping and to quantify peak intensity, resulting in a table of calibrated peak intensities to be matched with metabolites with the aid of spectral databases (steps 5–6); univariate and multivariate statistics lead to candidate biomarkers being correlated with the pathologic state by pathway analysis and lab testing.

**Figure 3:**
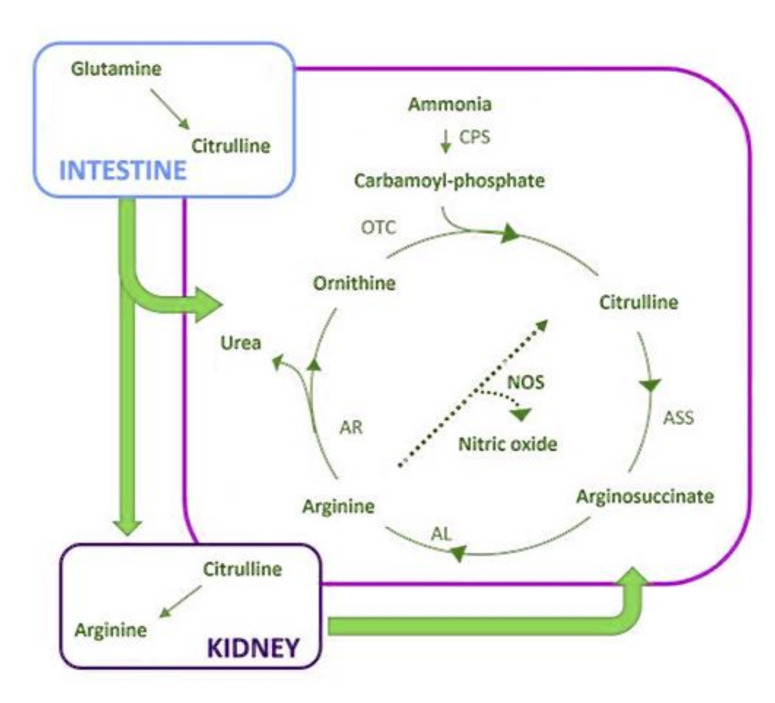
Decrease in citrulline level has been observed for many soft tissue sarcomas. Citrulline belongs to the arginine metabolic pathway: it is produced by enterocytes from glutamine and is released into the blood, where is taken up by the kidney for the synthesis of arginine or is transported to the liver where it participates in the transformation of ammonia to urea. Abbreviations: CPS, carbamoyl phosphate synthetase; ASS, argininosuccinate synthase; AL, argininosuccinate lyase; AR, arginase; OTC, ornithine transcarbamoylase.

A new approach in biomarker research is provided by metabolomics, an emerging omics science focused on analysis of the full set of metabolites present in a biological sample [137,138,139]. The metabolic profile of the organism under investigation describes the undergoing biochemical events and reflects the complex interactions among gene transcription, protein expression, and physio-pathological conditions, including gut microbiome activity and environmental effects [139,140]. A peculiarity of metabolomics is providing the functional readout of the phenotype from a single analysis run of a biofluid sample (urine, plasma, serum, saliva, cerebrospinal fluid) by exploiting the resolving power of instrumental techniques such as nuclear magnetic resonance (NMR) and mass spectrometry (MS), also in a high-throughput fashion [139]. Metabolite identification and quantification is powered by growing databases of molecular spectra, both in the free and commercial domain, e.g., HMDB [141], BMRB [142], SDBS [143], CHENOMX [144], BBIOREFCODE [145], and MetaboBASE [146], while the discovery of new small molecule biomarkers is possible thanks to the structure elucidation ability offered by multidimensional NMR [147,148] in combination with MS [149,150,151]. Metabolic profiles of different cohorts allow for the discrimination of pathologic state, comparison of treatment outcome and risk stratification, even in the absence of prior knowledge of relevant biomarkers as in untargeted metabolomics [152]. Not only the metabolites, but also full datasets from metabolomics studies have found a home in the MetaboLights database [153] (Figure 2). Although metabolomics studies have already been widely used to identify diagnostic and prognostic biomarkers in cancer [154,155,156,157,158], only a few have been reported for STS. One of the first metabolomic studies on STS was an innovative application of liquid chromatography tandem MS (LC/MS/MS) to formalin-fixed and paraffin-embedded (FFPE) tissue specimens acquired during routine medical care [159]. The study regarded one each of high-grade sarcoma with myogenic differentiation, high-grade leiomyosarcoma, monophasic synovial sarcoma, biphasic synovial sarcoma and well-differentiated liposarcoma. Eight distinct, differentially abundant metabolites between the STSs and control groups were found, but the significance of these results is hampered by the lack of comparison with fresh or frozen specimens. From this study onward, biofluids from clinical contexts or cell extracts from in vitro settings have been used. By using LC/MS/MS [160], nucleoside salvage pathway activity was observed in liposarcoma cell lines derived from xenograft tumors. The same cell lines were found to be sensitive to treatment with the nucleoside-based prodrug gemcitabine (which relies on nucleoside salvage activity), suggesting nucleosides as biomarkers to delineate gemcitabine responders from non-responders. In addition to nucleosides, the study also identified other metabolites consumed by liposarcoma cell lines, including amino acids and amino acid precursors. Particularly, high consumption of glutamine was reported as preliminary data, a finding that has been confirmed in a recent study from Lee et al. [161]. The study reports an in vitro investigation of glutamine, glutamate, aspartate, and asparagine metabolism by UPLC/MS analysis of tissue extracts from an undifferentiated pleomorphic sarcoma mouse model, showing how UPS relies on glutamine as a source of energy and biosynthetic anabolism. Glutamine is indeed an important nutrient for highly proliferative cells due to its support for cellular bio-energetics and bio-syntheses. In particular, glutamine’s carbon backbone is involved in the synthesis of tricarboxylic acid (TCA) cycle intermediates, amino acids, and other metabolites [162,163,164,165,166], whereas glutamine nitrogen also promotes nucleotide bio-synthesis [166]. The rate-limiting enzyme for glutaminolysis is glutaminase, which exists in two isoforms, glutaminase 1 (GLS1) and 2 (GLS2), with the former detected as the predominant isoform in murine tumors and cell lines. A metabolomic study on the role of glutamine [161] also led to testing of the drug Telaglenastat, currently in clinical trials for multiple cancer types; it was shown that GLS inhibition mimics glutamine starvation and is effective in causing UPS and additional STS cell death. Regarding leiomyosarcoma, Miolo et al. [137] used a metabolomics approach to search for new serum prognostic markers for overall survival in 24 patients with metastatic STS treated with trabectedin. Leiomyosarcoma was indeed the most prevalent histotype (*n* = 8, 33.3%) among the others (malignant peripheral nerve sheath tumor (*n* = 3), fibrosarcoma (*n* = 2), undifferentiated pleomorphic sarcoma (*n* = 2), chondrosarcoma (*n* = 2), synovial sarcoma (*n* = 2), not otherwise specified sarcoma (*n* = 2), endometrial stromal sarcoma (*n* = 1), and desmoplastic small round cell tumor (*n* = 1)). The study takes into account the serum levels of 68 targeted metabolites (53 amino acids and their derivatives plus 15 bile acids), determined by LC/MS/MS. The results indicate a citrulline shortage in high-risk patients, making this amino acid an important metabolic signature possibly explaining the high overall survival variability of STS patients. Citrulline belongs to the arginine metabolic pathway. It is produced by enterocytes from glutamine and released into the blood, where is taken up by the kidney for the synthesis of arginine or is transported to the liver where it participates in the transformation of ammonia to urea [167] (Figure 3). As already commented, a tumor is a high energy-demanding tissue that requires, besides glucose, other carbon intermediates, which may result in deficiencies of certain metabolites in the blood due to metabolic reprogramming of the whole organism [156], as is the case for the observed citrulline shortage, in line with the findings for glutamine metabolism in UPS [161]. In this view, an interesting result of the study is that the low citrulline levels were not associated with a lack of the precursor glutamine. An interesting case study from the same group highlighted a specific metabolic pattern for chemo-resistance phenotypes of a patient affected by STS with real-time analysis of breath-exhaled volatile organic compounds, performed by select ion flow tube MS (SIFT-MS) [168]. The study also highlighted how the high sensitivity of MS could lead to non-invasive, innovative metabolomic analyses such as breath analysis. A study with more heterogenous STS subtypes was conducted by Jia et al. [169]. The authors reported an LC/MS/MS investigation targeting plasma-free amino acid profiles (PFAAs) of 23 patients: there were six cases of myogenic sarcoma (26.1%), five cases of undifferentiated pleomorphic sarcoma (21.7%), two cases of liposarcoma (8.7%), two cases of acinar soft-tissue sarcoma (8.7%), and other types of sarcoma in one case each (4.3%), including chondrosarcoma, osteosarcoma, Ewing’s sarcoma, mucosarcoma, pulmonary artery sarcoma, endometrial stromal sarcoma, synovial sarcoma and fibrosarcoma. PFAAs have shown different features in various cancers, but the characteristic in STS is still unclear. Seven differential amino acids were identified: sarcosine (Sar), glutamine (Gln), homoproline (Hpro), citrulline (Cit) decreased, whereas carnosine (Car), lysine (Lys), glutamic acid (Glu) increased in the sarcoma patients. The increased Glu levels could be interpreted as a result of increased Gln metabolism in the tumor. Clustering into classes showed how total amino acids (TAAs), branched-chain amino acids (BCAAs), aromatic amino acids (AAAs) and glycemic amino acids (GAAs) decreased significantly, but essential amino acids (EAAs) increased significantly. Metabolic profiles allowed four pathways to be predicted as affected by sarcoma, with arginine biosynthesis at affected the most; such a finding, confirmed by other evidence, that Arg stimulates tumor growth [170], suggests arginine metabolic pathways as a potential targets in sarcoma, as also indicated by similar conclusions in the abovementioned study by Miolo et al. [137]. Moreover, the change of PFAAs after one chemotherapy cycle in sarcoma patients [169] showed that that levels of γ-aminobutyric acid (GABA) and Car decreased significantly in the improvement group but not in the deterioration group, whereas levels of α-aminobutyric acid (Abu) increased significantly in the deterioration group but not in the improvement group, suggesting the three as biomarkers for monitoring of the chemotherapy outcome. Focuses on other specific sarcoma subgroups are also found. One of the first extensive metabolic profiling studies of osteosarcoma [171] also involved benign bone tumors such as chondrosteoma, aneurysmal bone cyst, chondromyxoid fibroma, enchondroma, and osteofibrous dysplasia as references. The serum profile showed an increased concentration of cystine and 2-hydroxybutyrate and decreased levels of malate and dodecanoic acid for osteosarcoma as well as for the benign tumors, but significance for the latter was low because just one sample of each was used. The metabolic profile of cartilage tumors (CTs) was improved in an NMR study of serum from patients affected by enchondromas and chondrosarcomas (three each) [172], showing a strong association with the dysregulation of taurine and hypotaurine metabolism as well as the synthesis and degradation of ketone bodies.

## 4. Circulating Biomarkers

Novel circulating biomarker candidates such as tumor-derived extracellular vescicles (EVs) and circulating tumor cells (CTCs) have been studied in STS [173,174,175]. Even though data regarding the prognostic significance of CTC isolation in STS are limited, a correlation between CTC presence and disease progression has been observed [176,177]. The detection of CTCs in sarcomas has been proposed to lead to better understanding of the efficacy of therapy and of drug resistance [177,178]. The advent of new technologies to determine and monitor these biological entities in LB [179,180,181] holds great promise for developing minimally invasive methods to improve patient care.

## 5. Biomarkers in Clinical Trials

Clinical results on ICIs in STS have not shown strong improvements and trials have been slower in development, suggesting that more effort should be made to identify which patients are most likely to respond through predictive biomarker development [182]. Several trials of mono or combination checkpoint inhibitors have tried to identify the immune biomarkers expressed in STS that are relevant to clinical management and are summarized in Table 2 and Table 3, respectively. Bertucci et al. 2017 [183] showed that PDL1 mRNA expression is heterogenous in STS, and is an independent prognostic factor of metastatic relapse. Recently clinically evaluated transcriptomic biomarker signatures such as Complexity INdex in SARComas (CINSARC), genomic grade index and hypoxia-associated signature can be integrated with biomarkers of targeted therapy enhancing prognostication [173].

ICIs combined with TKIs have shown promise. A phase II trial enrolling patients with alveolar soft part sarcoma (ASPS), characterized by ASPSCR1–TFE3 fusion gene and consequently with an upregulation of vascular endothelial growth factor (VEGF), explores the combination of pembrolizumab with the VEGF receptor inhibitor, axitinib. Clinical benefit was observed in 73% of patients [184]. Atezolizumab, a monoclonal antibody against PD-L1, is currently under investigation in a phase II trial of unresectable ASPS (NCT03141684). The use of adoptive T cell transfer with enhanced affinity for tumor-specific antigens (such as New York esophageal squamous cell carcinoma-1 (NY-ESO-1) and melanoma antigen gene type A4 (MAGE-A4)) has also shown early promise in STS, particularly in synovial sarcoma [185].

Although STSs do not have a characterized defect in BRCA1/2, their genomics are complex in roughly 50% of cases, suggesting genomic instability and an eventual possible deficiency in DNA damage repair, as reported for leiomyosarcomas (Chudasama, 2018). Thus, STSs could be efficiently targeted with Poly(ADP-ribose) polymerase (PARP) inhibitors to drive cells to synthetic lethality (Table 4).

Recently, the safety of the combination of trabectedin chemotherapy and olaparib PARP inhibitor in second-line or further-line therapy has been shown in patients with advanced STS [186] with an 18% partial response. The response rate and progression-free survival were higher in patients with high *PARP1* tumor expression [187]. A retrospective series demonstrated that the expression of PARP1 and tumors expressing high levels had worse MFS [183]. In addition, it has been reported that PARP-1 expression complemented the prognostic value of CINSARC on 5-year metastasis free survival [173].

A phase II study evaluated tazemetostat, an EZH2 inhibitor, in patients with epithelioid sarcoma (which comprises <1% of STS), and the observed response rate registered was 15% and disease control rate was 26% [188]. Tazemetostat is currently under priority review with the FDA. The nonrandomized, open-label, registrational phase II AMPECT trial explored a novel nanoparticle albumin-bound (nab)-form of sirolimus in patients with unresectable malignant perivascular epithelioid cell tumors (characterized by dysregulation of the mammalian target of rapamycin (mTOR) pathway as a result of tuberous sclerosis 1 or 2 (TSC1 or TSC2) deletions/mutations), revealed a response rate of 42%, a disease control rate of 77%, and a median PFS of 8.9 months [189]. The dedifferentiated liposarcomas are characterized by mouse double minute 2 (MDM2) and cyclin-dependent kinase 4 (CDK4) amplifications, and the CDK4 inhibitor, abemaciclib, is currently under investigation; an encouraging 12-week PFS of 76% in a phase II nonrandomized trial has been reported [27,190] (Table 5).

Recently, some retrospective studies have indicated that targeted sequencing can be useful to hypothesize alternative treatment options [191,192]. The ongoing randomized, phase III MULTISARC clinical trial (NCT03784014) compares the standard of care therapy with the indications emerging from NGS to enroll patients into sub-arms of targeted therapies. 

Tumors harboring neurotrophic tyrosine receptor kinase (NTRK) fusions have shown durable responses to the tropomyosin receptor kinase (TRK) inhibitor, larotrectinib [193].

## 6. Limitations

The molecular biology of STS cannot be covered exhaustively within one single review, because STSs show tremendous heterogeneity both in clinical and genomic settings. Furthermore, extensive information is promptly accumulating contributing to the further understanding of the molecular biology of STS; there are, however, enormous challenges ahead, particularly in the clinical translation of these discoveries. In the current review, we have focused uniquely on the description of the established and most attractive biomarkers rather than their complex and multiple interactions; this needs an additional article to be adequately addressed. One of the limits of the current review could be the omission of some studies related to our topic due to the fact that STSs encompass a very heterogeneous group of tumors with diverse pathological and clinical overlapping characteristics.

Several novel and relevant biomarkers are emerging, but to achieve the necessary level of evidence for incorporation into international guidelines and use in the clinical setting, there is a need for additional prospective clinical trials.

## 7. Future Perspectives and Conclusions

Due to their rarity and heterogeneity, generating high-quality evidence for the management of STS is still challenging. Advances in research methodology and technology have helped in the exploration of pathogenetic mechanisms and histopathological characteristics of these tumors, but this has not yet translated into significant improvements in treatment strategies, and unfortunately results with survival rates have barely improved over the last three decades. There is, therefore, an urgent need to develop better prognostic and diagnostic tools so that appropriate measures can be taken in a histotype-specific and timely manner, especially in the case of advanced sarcomas. Identifying non-invasive, reliable biomarkers can represent a step towards improving the survival in STS both for an earlier diagnosis and for a tailored treatment selection with a ‘next generation’ approach for STS management. Selected biomarkers can offer an improvement not only for adjuvant therapy selection (radiotherapy, chemotherapy, immunotherapy), but also for surgical excision, which nowadays remains the mainstay of treatment for STSs, because biomarker-mediated imaging of STS during surgery has recently been proposed to facilitate complete resection by visualizing tumor tissue during surgery.

A wide quantity of contemporary data have been collected at molecular, metabolic and cellular level, and the research is still ongoing with a long path ahead. Correlations between laboratory and clinical data are still at the beginning. Will it be able to change our future treatment strategies in STSs?

## Figures and Tables

**Table 1:** (**A**). Main molecular markers associated with prognosis in STSs, (**B**). Main molecular markers associated with sensitivity/resistance to therapy in STSs.

**A. Main Molecular Markers Associated with Prognosis in STSs**		
**Molecular Marker**	**Prognosis**	**References**
Microenvironment		
high B cells	better survival	[24]
high CD8^+^ T cells	improvement in survival	[24,29,30,31,32]
low CD8^+^ T cells at relapse	poor survival	[18]
high CD4+ T cells	improvement in survival	[24,28]
low CD163^+^ TIMs	favorable survival	[15]
high levels CD163^+^	improvement in survival	[22]
PDL1^+^	worse prognosis	[39]
LAG3+ CD8^+^ T cells	severe prognosis	[45]
LAG3+ CD4^+^ T cells	severe prognosis	[45]
Genetics		
miR26a-2c amplification	poor survival	[95]
high expression of miR-17-92 cluster	poor prognosis	[101]
miRNA panel *	predicted in metastatic events	[108]
miR-138 overexpression	negative correlation with MFS	[116]
miR-210	severe prognosis	[121]
LncRNAs: LINC00680, AC006129.2, RP11-274B21.9 and RP11-713P17.3	favorable prognosis	[124]
LncRNAs: RP11-560J1.2, AP001432.14, RP4-665J23.1, RP11-230G5.2, BACH1-IT2, and RP11504A18.1	severe prognosis	[124]
citrulline shortage	poor outcome	[137]
**B. Main Molecular Markers Associated with Sensitivity/Resistance to Therapy in STSs.**		
**Molecular Marker**	**Sensitivity/Resistance to Therapy**	**References**
CD8^+^ and CD4^+^ T cells	response to therapies	[24]
Alterations in CD68^+^, CD163^+^	neoadjuvant chemotherapy	[34]
increase in PDL1 levels	radiotherapy	[40]
IDO1/KP signaling pathway	resistance to PD1 inhibitors	[42]
miR-485-3p–Top2α–NF-YB pathway	sensitivity to therapies	[101,102]

Note. * miR-99a-5p, miR-146b-5p, miR-148b-3p, miR-195-5p, miR-223-3p, miR-500b-3p, miR-505-3p.

**Table 2:** Selected immunotherapy trials in STS ^a^.

Immune Checkpoint Inhibitor	Trial Number	Phase	Status
Ipilimumab (anti-CTLA4)	NCT01445379	I	Completed
Pembrolizumab (anti-PD1)	NCT02301039NCT03316573	II II	Completed Recruiting
Toripalimab (anti-PD1)	NCT03474640	I	Recruiting
Spartalizumab (anti-PD1)	NCT04802876	II	Not yet recruiting

Note. ^a^ Source: clinicaltrials.gov (accessed on 14 May 2021).

**Table 3:** Selected immunotherapy combination trials in STS ^a^.

Immune Checkpoint Inhibitors	Other Agents	Trial Number	Phase	Status
Immune checkpoint inhibitors in combination with other immunotherapy agents				
Pembrolizumab	IDO 1 Inhibitor (i) (epacadostat)	NCT03414229	II	Active, not recruiting
Nivolumab (anti-PD1)	Ipilimumab (anti-CTLA4)	NCT04741438	III	Recruiting
	Relatlimab (anti-LAG3)	NCT04095208	II	Recruiting
	NKTR-214	NCT03282344	II	Active, not recruiting
Immune checkpoint inhibitors in combination with chemotherapy or radiation therapy				
Atezolizumab (anti-PDL1)	Radiation therapy	NCT03474094	II	Recruiting
Nivolumab	Paclitaxel	NCT04339738	II	Recruiting
	Trabectedin	NCT03590210	II	Active, not recruiting
	T-VEC and Trabectedin	NCT03886311	II	Recruiting
Pembrolizumab	Radiation therapy	NCT03338959	I-II	Recruiting
	Eribuline	NCT03899805	II	Active, not recruiting
	T-VEC	NCT03069378	II	Recruiting
	Lenvatinib (VEGFR/FGFR-i)	NCT04784247	II	Recruiting
	Axitinib (VEGFR- i)	NCT02636725	II	Active, not recruiting
Immune checkpoint inhibitors in combination with other agents				
Atezolizumab	Bevacizumab (VEGF-i)	NCT03141684	II	Recruiting
	Rucaparib (PARP-i)	NCT04216953	I-II	Recruiting
	Cobimetinib (MEK-i)	NCT04624178	II	Recruiting
Dual immune checkpoint inhibitors therapy in combination with other agents				
Nivolumab plus ipilimumab	Cabozantinib (VEGFR/MET/AXL-i)	NCT04551430	II	Recruiting
	Trabectedin	NCT03138161	I-II	Recruiting

Note. ^a^ Source: clinicaltrials.gov (accessed on 14 May 2021).

**Table 4:** Selected PARP-inhibitor trials in sarcoma ^a.^

PARP-Inhibitor	Other Agents	NCT Trial Number	Phase	Status
Olaparib	Radiation therapy	NCT02787642	I	Recruiting
Olaparib	Temozolomide	NCT01858168	I	Recruiting
Olaparib	Durvalumab	NCT03784014	III	Recruiting
Olaparib	Trabectedin	NCT04076579	II	Recruiting
Olaparib	Pembrolizumab	NCT04123366	II	Recruiting
Olaparib	-	NCT03233204	II	Recruiting
Rucaparib	-	NCT04171700	II	Recruiting

Note. ^a^ Source: clinicaltrials.gov (accessed on 14 May 2021).

**Table 5:** Selected multitargeted TKI-i trials in sarcoma ^a^.

Multitargeted TKI-i	Other Agents	NCT Trial Number	Phase	Status
EZH2 inhibitors (tazemetostat)	Doxorubicin	NCT04204941	III	Recruiting
Nab-rapamycin	Nivolumab	NCT03190174	I-II	Recruiting
TRK inhibitor (Larotrectinib)	-	NCT02576431	II	Recruiting
CDK4 inhibitors (Abemaciclib)	-	NCT04040205	II	Recruiting
	Temozolomide and Irinotecan	NCT04238819	I	Recruiting

Note. ^a^ Source: clinicaltrials.gov (accessed on 14 May 2021).

## References

[1] International Agency for Research on Cancer (2020). WHO Classification of Tumours Editorial Board. Soft Tissue and Bone Tumours.

[2] Choi J.H., Ro J.Y. (2021). The 2020 WHO Classification of Tumors of Soft Tissue: Selected Changes and New Entities. Adv. Anat. Pathol..

[3] Trama A., Badalamenti G., Baldi G.G., Brunello A., Caira M., Drove N., Marrari A., Palmerini E., Vincenzi B., Dei Tos A.P. (2019). Soft tissue sarcoma in Italy: From epidemiological data to clinical networking to improve patient care and outcomes. Cancer Epidemiol..

[4] Ferrari A., Sultan I., Huang T.T., Rodriguez-Galindo C., Shehadeh A., Meazza C., Ness K.K., Casanova M., Spunt S.L. (2011). Soft tissue sarcoma across the age spectrum: A population-based study from the Surveillance Epidemiology and End Results database. Pediatr. Blood Cancer.

[5] Blay J.-Y., Honoré C., Stoeckle E., Meeus P., Jafari M., Gouin F., Anract P., Ferron G., Rochwerger A., Ropars M. (2019). Surgery in reference centers improves survival of sarcoma patients: A nationwide study. Ann. Oncol. Off. J. Eur. Soc. Med. Oncol..

[6] Kleinerman R.A., Tucker M.A., Abramson D.H., Seddon J.M., Tarone R.E., Fraumeni J.F. (2007). Risk of Soft Tissue Sarcomas by Individual Subtype in Survivors of Hereditary Retinoblastoma. JNCI J. Natl. Cancer Inst..

[7] Italiano A., Bringer S., Blay J.-Y., Bonvalot S., Le Cesne A., Le Loarer F., Maingon P. (2019). Patterns of Care and Outcome Radiation-Induced Soft Tissue Sarcomas. Int. J. Radiat. Oncol. Biol. Phys..

[8] McClain K.L., Leach C.T., Jenson H.B., Joshi V.V., Pollock B.H., Parmley R.T., DiCarlo F.J., Chadwick E.G., Murphy S.B. (1995). Association of Epstein-Barr virus with leiomyosarcomas in young people with AIDS. N. Engl. J. Med..

[9] Viel J.F., Arveux P., Baverel J., Cahn J.Y. (2000). Soft-tissue sarcoma and non-Hodgkin’s lymphoma clusters around a municipal solid waste incinerator with high dioxin emission levels. Am. J. Epidemiol..

[10] Zambon P., Ricci P., Bovo E., Casula A., Gattolin M., Fiore A.R., Chiosi F., Guzzinati S. (2007). Sarcoma risk and dioxin emissions from incinerators and industrial plants: A population-based case-control study (Italy). Environ. Health.

[11] Kabir W., Choong P.F.M., Choong P.F.M. (2021). The Epidemiology and Pathogenesis of Sarcoma BT—Sarcoma: A Practical Guide to Multidisciplinary Management.

[12] Casali P.G., Abecassis N., Aro H.T., Bauer S., Biagini R., Bielack S., Bonvalot S., Boukovinas I., Bovee J.V.M.G., Brodowicz T. (2018). Soft tissue and visceral sarcomas: ESMO-EURACAN Clinical Practice Guidelines for diagnosis, treatment and follow-up. Ann. Oncol. Off. J. Eur. Soc. Med. Oncol..

[13] Abarca T., Gao Y., Monga V., Tanas M.R., Milhem M.M., Miller B.J. (2018). Improved survival for extremity soft tissue sarcoma treated in high-volume facilities. J. Surg. Oncol..

[14] Sarcoma Meta-Analysis Collaboration (1997). Adjuvant chemotherapy for localised resectable soft-tissue sarcoma of adults: Meta-analysis of individual data. Lancet.

[15] Pervaiz N., Colterjohn N., Farrokhyar F., Tozer R., Figueredo A., Ghert M. (2008). A systematic meta-analysis of randomized controlled trials of adjuvant chemotherapy for localized resectable soft-tissue sarcoma. Cancer.

[16] Gamboa A.C., Gronchi A., Cardona K. (2020). Soft-tissue sarcoma in adults: An update on the current state of histiotype-specific management in an era of personalized medicine. CA. Cancer J. Clin..

[17] Sharonov G.V., Serebrovskaya E.O., Yuzhakova D.V., Britanova O.V., Chudakov D.M. (2020). B cells, plasma cells and antibody repertoires in the tumour microenvironment. Nat. Rev. Immunol..

[18] Sorbye S.W., Kilvaer T., Valkov A., Donnem T., Smeland E., Al-Shibli K., Bremnes R.M., Busund L.-T. (2011). Prognostic impact of lymphocytes in soft tissue sarcomas. PLoS ONE.

[19] Tsagozis P., Augsten M., Zhang Y., Li T., Hesla A., Bergh J., Haglund F., Tobin N.P., Ehnman M. (2019). An immunosuppressive macrophage profile attenuates the prognostic impact of CD20-positive B cells in human soft tissue sarcoma. Cancer Immunol. Immunother..

[20] Lee A.T.J., Chew W., Wilding C.P., Guljar N., Smith M.J., Strauss D.C., Fisher C., Hayes A.J., Judson I., Thway K. (2019). The adequacy of tissue microarrays in the assessment of inter- and intra-tumoural heterogeneity of infiltrating lymphocyte burden in leiomyosarcoma. Sci. Rep..

[21] Yan L., Wang Z., Cui C., Guan X., Dong B., Zhao M., Wu J., Tian X., Hao C. (2019). Comprehensive immune characterization and T-cell receptor repertoire heterogeneity of retroperitoneal liposarcoma. Cancer Sci..

[22] Kather J.N., Hörner C., Weis C.-A., Aung T., Vokuhl C., Weiss C., Scheer M., Marx A., Simon-Keller K. (2019). CD163+ immune cell infiltrates and presence of CD54+ microvessels are prognostic markers for patients with embryonal rhabdomyosarcoma. Sci. Rep..

[23] Petitprez F., de Reyniès A., Keung E.Z., Chen T.W.-W., Sun C.-M., Calderaro J., Jeng Y.-M., Hsiao L.-P., Lacroix L., Bougoüin A. (2020). B cells are associated with survival and immunotherapy response in sarcoma. Nature.

[24] Jochems C., Schlom J. (2011). Tumor-infiltrating immune cells and prognosis: The potential link between conventional cancer therapy and immunity. Exp. Biol. Med..

[25] Martin F., Ladoire S., Mignot G., Apetoh L., Ghiringhelli F. (2010). Human FOXP3 and cancer. Oncogene.

[26] Budczies J., Mechtersheimer G., Denkert C., Klauschen F., Mughal S.S., Chudasama P., Bockmayr M., Jöhrens K., Endris V., Lier A. (2017). PD-L1 (CD274) copy number gain, expression, and immune cell infiltration as candidate predictors for response to immune checkpoint inhibitors in soft-tissue sarcoma. Oncoimmunology.

[27] Zhu M.M.T., Shenasa E., Nielsen T.O. (2020). Sarcomas: Immune biomarker expression and checkpoint inhibitor trials. Cancer Treat. Rev..

[28] Zheng B., Wang J., Cai W., Lao I., Shi Y., Luo X., Yan W. (2019). Changes in the tumor immune microenvironment in resected recurrent soft tissue sarcomas. Ann. Transl. Med..

[29] Van Erp A.E.M., Versleijen-Jonkers Y.M.H., Hillebrandt-Roeffen M.H.S., van Houdt L., Gorris M.A.J., van Dam L.S., Mentzel T., Weidema M.E., Savci-Heijink C.D., Desar I.M.E. (2017). Expression and clinical association of programmed cell death-1, programmed death-ligand-1 and CD8(+) lymphocytes in primary sarcomas is subtype dependent. Oncotarget.

[30] Oike N., Kawashima H., Ogose A., Hotta T., Hatano H., Ariizumi T., Sasaki T., Yamagishi T., Umezu H., Endo N. (2018). Prognostic impact of the tumor immune microenvironment in synovial sarcoma. Cancer Sci..

[31] Berghuis D., Santos S.J., Baelde H.J., Taminiau A.H., Egeler R.M., Schilham M.W., Hogendoorn P.C., Lankester A.C. (2011). Pro-inflammatory chemokine-chemokine receptor interactions within the Ewing sarcoma microenvironment determine CD8(+) T-lymphocyte infiltration and affect tumour progression. J. Pathol..

[32] Fujii H., Arakawa A., Utsumi D., Sumiyoshi S., Yamamoto Y., Kitoh A., Ono M., Matsumura Y., Kato M., Konishi K. (2014). CD8^+^ tumor-infiltrating lymphocytes at primary sites as a possible prognostic factor of cutaneous angiosarcoma. Int. J. Cancer.

[33] Klaver Y., Rijnders M., Oostvogels A., Wijers R., Smid M., Grünhagen D., Verhoef C., Sleijfer S., Lamers C., Debets R. (2020). Differential quantities of immune checkpoint-expressing CD8 T cells in soft tissue sarcoma subtypes. J. Immunother. Cancer.

[34] Raj S.K.S., Kooshki M., Winters M., Russell G.B., Miller L.D., Laurini J.A., Pierre T., Savage P.D. (2019). Prognostic implications of tumor associated macrophages (TAMs) in soft tissue sarcoma. J. Clin. Oncol..

[35] Kuol N., Stojanovska L., Nurgali K., Apostolopoulos V. (2018). PD-1/PD-L1 in disease. Immunotherapy.

[36] Zheng B., Ren T., Huang Y., Sun K., Wang S., Bao X., Liu K., Guo W. (2018). PD-1 axis expression in musculoskeletal tumors and antitumor effect of nivolumab in osteosarcoma model of humanized mouse. J. Hematol. Oncol..

[37] Torabi A., Amaya C.N., Wians F.H.J., Bryan B.A. (2017). PD-1 and PD-L1 expression in bone and soft tissue sarcomas. Pathology.

[38] Kim J.R., Moon Y.J., Kwon K.S., Bae J.S., Wagle S., Kim K.M., Park H.S., Lee H., Moon W.S., Chung M.J. (2013). Tumor infiltrating PD1-positive lymphocytes and the expression of PD-L1 predict poor prognosis of soft tissue sarcomas. PLoS ONE.

[39] Kim C., Kim E.K., Jung H., Chon H.J., Han J.W., Shin K.-H., Hu H., Kim K.S., Choi Y.D., Kim S. (2016). Prognostic implications of PD-L1 expression in patients with soft tissue sarcoma. BMC Cancer.

[40] Patel K.R., Martinez A., Stahl J.M., Logan S.J., Perricone A.J., Ferris M.J., Buchwald Z.S., Chowdhary M., Delman K.A., Monson D.K. (2018). Increase in PD-L1 expression after pre-operative radiotherapy for soft tissue sarcoma. Oncoimmunology.

[41] Munn D.H., Sharma M.D., Hou D., Baban B., Lee J.R., Antonia S.J., Messina J.L., Chandler P., Koni P.A., Mellor A.L. (2004). Expression of indoleamine 2,3-dioxygenase by plasmacytoid dendritic cells in tumor-draining lymph nodes. J. Clin. Investig..

[42] Nafia I., Toulmonde M., Bortolotto D., Chaibi A., Bodet D., Rey C., Velasco V., Larmonier C.B., Cerf L., Adam J. (2020). IDO Targeting in Sarcoma: Biological and Clinical Implications. Front. Immunol..

[43] Friedlaender A., Addeo A., Banna G. (2019). New emerging targets in cancer immunotherapy: The role of TIM3. ESMO Open.

[44] Solinas C., Migliori E., De Silva P., Willard-Gallo K. (2019). LAG3: The Biological Processes That Motivate Targeting This Immune Checkpoint Molecule in Human Cancer. Cancers.

[45] Que Y., Fang Z., Guan Y., Xiao W., Xu B., Zhao J., Chen H., Zhang X., Zeng M., Liang Y. (2019). LAG-3 expression on tumor-infiltrating T cells in soft tissue sarcoma correlates with poor survival. Cancer Biol. Med..

[46] Feng M., Jiang W., Kim B.Y.S., Zhang C.C., Fu Y.-X., Weissman I.L. (2019). Phagocytosis checkpoints as new targets for cancer immunotherapy. Nat. Rev. Cancer.

[47] Dancsok A.R., Gao D., Lee A.F., Steigen S.E., Blay J.-Y., Thomas D.M., Maki R.G., Nielsen T.O., Demicco E.G. (2020). Tumor-associated macrophages and macrophage-related immune checkpoint expression in sarcomas. Oncoimmunology.

[48] Goodman A.M., Kato S., Bazhenova L., Patel S.P., Frampton G.M., Miller V., Stephens P.J., Daniels G.A., Kurzrock R. (2017). Tumor Mutational Burden as an Independent Predictor of Response to Immunotherapy in Diverse Cancers. Mol. Cancer Ther..

[49] (2017). Comprehensive and Integrated Genomic Characterization of Adult Soft Tissue Sarcomas. Cell.

[50] Xu L.-B., Zhao Z.-G., Xu S.-F., Zhang X.-X., Liu T., Jing C.-Y., Zhang S.-G., Yu S.-J. (2020). The landscape of gene mutations and clinical significance of tumor mutation burden in patients with soft tissue sarcoma who underwent surgical resection and received conventional adjuvant therapy. Int. J. Biol. Markers.

[51] Chalmers Z.R., Connelly C.F., Fabrizio D., Gay L., Ali S.M., Ennis R., Schrock A., Campbell B., Shlien A., Chmielecki J. (2017). Analysis of 100,000 human cancer genomes reveals the landscape of tumor mutational burden. Genome Med..

[52] He M., Abro B., Kaushal M., Chen L., Chen T., Gondim M., Yan W., Neidich J., Dehner L.P., Pfeifer J.D. (2020). Tumor mutation burden and checkpoint immunotherapy markers in primary and metastatic synovial sarcoma. Hum. Pathol..

[53] Joseph C.G., Hwang H., Jiao Y., Wood L.D., Kinde I., Wu J., Mandahl N., Luo J., Hruban R.H., Diaz L.A.J. (2014). Exomic analysis of myxoid liposarcomas, synovial sarcomas, and osteosarcomas. Genes. Chromosomes Cancer.

[54] Agelopoulos K., Richter G.H.S., Schmidt E., Dirksen U., von Heyking K., Moser B., Klein H.-U., Kontny U., Dugas M., Poos K. (2015). Deep Sequencing in Conjunction with Expression and Functional Analyses Reveals Activation of FGFR1 in Ewing Sarcoma. Clin. Cancer Res. Off. J. Am. Assoc. Cancer Res..

[55] Wooster R., Cleton-Jansen A.M., Collins N., Mangion J., Cornelis R.S., Cooper C.S., Gusterson B.A., Ponder B.A., von Deimling A., Wiestler O.D. (1994). Instability of short tandem repeats (microsatellites) in human cancers. Nat. Genet..

[56] Groisberg R., Roszik J., Conley A.P., Lazar A.J., Portal D.E., Hong D.S., Naing A., Herzog C.E., Somaiah N., Zarzour M.A. (2020). Genomics, Morphoproteomics, and Treatment Patterns of Patients with Alveolar Soft Part Sarcoma and Response to Multiple Experimental Therapies. Mol. Cancer Ther..

[57] Miquel C., Jacob S., Grandjouan S., Aimé A., Viguier J., Sabourin J.-C., Sarasin A., Duval A., Praz F. (2007). Frequent alteration of DNA damage signalling and repair pathways in human colorectal cancers with microsatellite instability. Oncogene.

[58] Sinicrope F.A., Sargent D.J. (2012). Molecular pathways: Microsatellite instability in colorectal cancer: Prognostic, predictive, and therapeutic implications. Clin. Cancer Res. Off. J. Am. Assoc. Cancer Res..

[59] Rucińska M., Kozłowski L., Pepiński W., Skawrońska M., Janica J., Wojtukiewic M.Z. (2005). High grade sarcomas are associated with microsatellite instability (chromosom 12) and loss of heterozygosity (chromosom 2). Med. Sci. Monit. Int. Med. J. Exp. Clin. Res..

[60] Kawaguchi K.-I., Oda Y., Takahira T., Saito T., Yamamoto H., Kobayashi C., Tamiya S., Oda S., Iwamoto Y., Tsuneyoshi M. (2005). Microsatellite instability and hMLH1 and hMSH2 expression analysis in soft tissue sarcomas. Oncol. Rep..

[61] Campanella N.C., Penna V., Ribeiro G., Abrahão-Machado L.F., Scapulatempo-Neto C., Reis R.M. (2015). Absence of Microsatellite Instability in Soft Tissue Sarcomas. Pathobiology.

[62] Ranjan A., Iwakuma T. (2016). Non-Canonical Cell Death Induced by p53. Int. J. Mol. Sci..

[63] Parrales A., Iwakuma T. (2015). Targeting Oncogenic Mutant p53 for Cancer Therapy. Front. Oncol..

[64] Oliner J.D., Pietenpol J.A., Thiagalingam S., Gyuris J., Kinzler K.W., Vogelstein B. (1993). Oncoprotein MDM2 conceals the activation domain of tumour suppressor p53. Nature.

[65] Mariño-Enríquez A., Bovée J.V.M.G. (2016). Molecular Pathogenesis and Diagnostic, Prognostic and Predictive Molecular Markers in Sarcoma. Surg. Pathol. Clin..

[66] Ognjanovic S., Olivier M., Bergemann T.L., Hainaut P. (2012). Sarcomas in TP53 germline mutation carriers: A review of the IARC TP53 database. Cancer.

[67] Nevins J.R. (2001). The Rb/E2F pathway and cancer. Hum. Mol. Genet..

[68] Kleinerman R.A., Schonfeld S.J., Tucker M.A. (2012). Sarcomas in hereditary retinoblastoma. Clin. Sarcoma Res..

[69] Korf B.R. (2013). Neurofibromatosis. Handb. Clin. Neurol..

[70] Brems H., Beert E., de Ravel T., Legius E. (2009). Mechanisms in the pathogenesis of malignant tumours in neurofibromatosis type 1. Lancet. Oncol..

[71] Rodon J., Dienstmann R., Serra V., Tabernero J. (2013). Development of PI3K inhibitors: Lessons learned from early clinical trials. Nat. Rev. Clin. Oncol..

[72] Movva S., Wen W., Chen W., Millis S.Z., Gatalica Z., Reddy S., von Mehren M., Van Tine B.A. (2015). Multi-platform profiling of over 2000 sarcomas: Identification of biomarkers and novel therapeutic targets. Oncotarget.

[73] Yin L., Liu C.X., Nong W.X., Chen Y.Z., Qi Y., Li H.A., Hu W.H., Sun K., Li F. (2012). Mutational analysis of p53 and PTEN in soft tissue sarcoma. Mol. Med. Rep..

[74] Kourea H.P., Orlow I., Scheithauer B.W., Cordon-Cardo C., Woodruff J.M. (1999). Deletions of the INK4A gene occur in malignant peripheral nerve sheath tumors but not in neurofibromas. Am. J. Pathol..

[75] Berner J.M., Sørlie T., Mertens F., Henriksen J., Saeter G., Mandahl N., Brøgger A., Myklebost O., Lothe R.A. (1999). Chromosome band 9p21 is frequently altered in malignant peripheral nerve sheath tumors: Studies of CDKN2A and other genes of the pRB pathway. Genes Chromosomes Cancer.

[76] Modena P., Lualdi E., Facchinetti F., Galli L., Teixeira M.R., Pilotti S., Sozzi G. (2005). SMARCB1/INI1 tumor suppressor gene is frequently inactivated in epithelioid sarcomas. Cancer Res..

[77] Davicioni E., Finckenstein F.G., Shahbazian V., Buckley J.D., Triche T.J., Anderson M.J. (2006). Identification of a PAX-FKHR gene expression signature that defines molecular classes and determines the prognosis of alveolar rhabdomyosarcomas. Cancer Res..

[78] Romualdi C., De Pittà C., Tombolan L., Bortoluzzi S., Sartori F., Rosolen A., Lanfranchi G. (2006). Defining the gene expression signature of rhabdomyosarcoma by meta-analysis. BMC Genom..

[79] Skapek S.X., Ferrari A., Gupta A.A., Lupo P.J., Butler E., Shipley J., Barr F.G., Hawkins D.S. (2019). Rhabdomyosarcoma. Nat. Rev. Dis. Prim..

[80] Nielsen T.O., West R.B., Linn S.C., Alter O., Knowling M.A., O’Connell J.X., Zhu S., Fero M., Sherlock G., Pollack J.R. (2002). Molecular characterisation of soft tissue tumours: A gene expression study. Lancet.

[81] Allander S.V., Illei P.B., Chen Y., Antonescu C.R., Bittner M., Ladanyi M., Meltzer P.S. (2002). Expression profiling of synovial sarcoma by cDNA microarrays: Association of ERBB2, IGFBP2, and ELF3 with epithelial differentiation. Am. J. Pathol..

[82] Panagopoulos I., Mertens F., Isaksson M., Limon J., Gustafson P., Skytting B., Akerman M., Sciot R., Dal Cin P., Samson I. (2001). Clinical impact of molecular and cytogenetic findings in synovial sarcoma. Genes Chromosomes Cancer.

[83] Shapiro D.N., Sublett J.E., Li B., Downing J.R., Naeve C.W. (1993). Fusion of PAX3 to a Member of the Forkhead Family of Transcription Factors in Human Alveolar Rhabdomyosarcoma. Cancer Res..

[84] Sorensen P.H.B., Lynch J.C., Qualman S.J., Tirabosco R., Lim J.F., Maurer H.M., Bridge J.A., Crist W.M., Triche T.J. (2002). Barr, alveolar rhabdomyosarcoma: A report from F.G. PAX3-FKHR and PAX7-FKHR gene fusions are prognostic indicators in the children’s oncology group. J. Clin. Oncol. Off. J. Am. Soc. Clin. Oncol..

[85] Xie Z., Babiceanu M., Kumar S., Jia Y., Qin F., Barr F.G., Li H. (2016). Fusion transcriptome profiling provides insights into alveolar rhabdomyosarcoma. Proc. Natl. Acad. Sci. USA.

[86] Xie Z., Tang Y., Su X., Cao J., Zhang Y., Li H. (2019). PAX3-FOXO1 escapes miR-495 regulation during muscle differentiation. RNA Biol..

[87] Vorburger S.A., Hunt K.K., Pollock R.E. (2002). Experimental Approaches.

[88] Jour G., Scarborough J.D., Jones R.L., Loggers E., Pollack S.M., Pritchard C.C., Hoch B.L. (2014). Molecular profiling of soft tissue sarcomas using next-generation sequencing: A pilot study toward precision therapeutics. Hum. Pathol..

[89] Ugras S., Brill E., Jacobsen A., Hafner M., Socci N.D., Decarolis P.L., Khanin R., O’Connor R., Mihailovic A., Taylor B.S. (2011). Small RNA sequencing and functional characterization reveals MicroRNA-143 tumor suppressor activity in liposarcoma. Cancer Res..

[90] Renner M., Czwan E., Hartmann W., Penzel R., Brors B., Eils R., Wardelmann E., Büttner R., Lichter P., Schirmacher P. (2012). MicroRNA profiling of primary high-grade soft tissue sarcomas. Genes Chromosomes Cancer.

[91] Taylor B.S., DeCarolis P.L., Angeles C.V., Brenet F., Schultz N., Antonescu C.R., Scandura J.M., Sander C., Viale A.J., Socci N.D. (2011). Frequent alterations and epigenetic silencing of differentiation pathway genes in structurally rearranged liposarcomas. Cancer Discov..

[92] Fricke A., Cimniak A.F.V., Ullrich P.V., Becherer C., Bickert C., Pfeifer D., Heinz J., Stark G.B., Bannasch H., Braig D. (2018). Whole blood miRNA expression analysis reveals miR-3613-3p as a potential biomarker for dedifferentiated liposarcoma. Cancer Biomark..

[93] Zhang P., Bill K., Liu J., Young E., Peng T., Bolshakov S., Hoffman A., Song Y., Demicco E.G., Terrada D.L. (2012). MiR-155 is a liposarcoma oncogene that targets casein kinase-1α and enhances β-catenin signaling. Cancer Res..

[94] Borjigin N., Ohno S., Wu W., Tanaka M., Suzuki R., Fujita K., Takanashi M., Oikawa K., Goto T., Motoi T. (2012). TLS-CHOP represses miR-486 expression, inducing upregulation of a metastasis regulator PAI-1 in human myxoid liposarcoma. Biochem. Biophys. Res. Commun..

[95] Lee D.H., Amanat S., Goff C., Weiss L.M., Said J.W., Doan N.B., Sato-Otsubo A., Ogawa S., Forscher C., Koeffler H.P. (2013). Overexpression of miR-26a-2 in human liposarcoma is correlated with poor patient survival. Oncogenesis.

[96] Ciarapica R., Russo G., Verginelli F., Raimondi L., Donfrancesco A., Rota R., Giordano A. (2009). Deregulated expression of miR-26a and Ezh2 in rhabdomyosarcoma. Cell Cycle.

[97] Subramanian S., Lui W.O., Lee C.H., Espinosa I., Nielsen T.O., Heinrich M.C., Corless C.L., Fire A.Z., van de Rijn M. (2008). MicroRNA expression signature of human sarcomas. Oncogene.

[98] Missiaglia E., Shepherd C.J., Patel S., Thway K., Pierron G., Pritchard-Jones K., Renard M., Sciot R., Rao P., Oberlin O. (2010). MicroRNA-206 expression levels correlate with clinical behaviour of rhabdomyosarcomas. Br. J. Cancer.

[99] Armeanu-Ebinger S., Herrmann D., Bonin M., Leuschner I., Warmann S.W., Fuchs J., Seitz G. (2012). Differential expression of miRNAs in rhabdomyosarcoma and malignant rhabdoid tumor. Exp. Cell Res..

[100] Li L., Sarver A.L., Alamgir S., Subramanian S. (2012). Downregulation of microRNAs miR-1, -206 and -29 stabilizes PAX3 and CCND2 expression in rhabdomyosarcoma. Lab. Investig..

[101] Reichek J.L., Duan F., Smith L.M., Gustafson D.M., O’Connor R.S., Zhang C., Dunlevy M.J., Gastier-Foster J.M., Barr F.G. (2011). Genomic and clinical analysis of amplification of the 13q31 chromosomal region in alveolar rhabdomyosarcoma: A report from the Children’s Oncology Group. Clin. Cancer Res. Off. J. Am. Assoc. Cancer Res..

[102] Chen C.-F., He X., Arslan A.D., Mo Y.-Y., Reinhold W.C., Pommier Y., Beck W.T. (2011). Novel regulation of nuclear factor-YB by miR-485-3p affects the expression of DNA topoisomerase IIα and drug responsiveness. Mol. Pharmacol..

[103] Miyachi M., Tsuchiya K., Yoshida H., Yagyu S., Kikuchi K., Misawa A., Iehara T., Hosoi H. (2010). Circulating muscle-specific microRNA, miR-206, as a potential diagnostic marker for rhabdomyosarcoma. Biochem. Biophys. Res. Commun..

[104] Subramanian S., Thayanithy V., West R.B., Lee C.-H., Beck A.H., Zhu S., Downs-Kelly E., Montgomery K., Goldblum J.R., Hogendoorn P.C.W. (2010). Genome-wide transcriptome analyses reveal p53 inactivation mediated loss of miR-34a expression in malignant peripheral nerve sheath tumours. J. Pathol..

[105] Nuovo G.J., Schmittgen T.D. (2008). Benign metastasizing leiomyoma of the lung: Clinicopathologic, immunohistochemical, and micro-RNA analyses. Diagn. Mol. Pathol..

[106] Guled M., Pazzaglia L., Borze I., Mosakhani N., Novello C., Benassi M.S., Knuutila S. (2014). Differentiating soft tissue leiomyosarcoma and undifferentiated pleomorphic sarcoma: A miRNA analysis. Genes Chromosomes Cancer.

[107] Sarver A.L., Phalak R., Thayanithy V., Subramanian S. (2010). S-MED: Sarcoma microRNA expression database. Lab. Invest..

[108] Fricke A., Ullrich P.V., Heinz J., Pfeifer D., Scholber J., Herget G.W., Hauschild O., Bronsert P., Stark G.B., Bannasch H. (2015). Identification of a blood-borne miRNA signature of synovial sarcoma. Mol. Cancer.

[109] Presneau N., Eskandarpour M., Shemais T., Henderson S., Halai D., Tirabosco R., Flanagan A.M. (2013). MicroRNA profiling of peripheral nerve sheath tumours identifies miR-29c as a tumour suppressor gene involved in tumour progression. Br. J. Cancer.

[110] Lee Y.-B., Bantounas I., Lee D.-Y., Phylactou L., Caldwell M.A., Uney J.B. (2009). Twist-1 regulates the miR-199a/214 cluster during development. Nucleic Acids Res..

[111] Gong M., Ma J., Li M., Zhou M., Hock J.M., Yu X. (2012). MicroRNA-204 critically regulates carcinogenesis in malignant peripheral nerve sheath tumors. Neuro-Oncology.

[112] Zhang P., Garnett J., Creighton C.J., Al Sannaa G.A., Igram D.R., Lazar A., Liu X., Liu C., Pollock R.E. (2014). EZH2-miR-30d-KPNB1 pathway regulates malignant peripheral nerve sheath tumour cell survival and tumourigenesis. J. Pathol..

[113] Italiano A., Thomas R., Breen M., Zhang L., Crago A.M., Singer S., Khanin R., Maki R.G., Mihailovic A., Hafner M. (2012). The miR-17-92 cluster and its target THBS1 are differentially expressed in angiosarcomas dependent on MYC amplification. Genes Chromosomes Cancer.

[114] Kim J.H., Jeon S., Shin B.A. (2017). MicroRNA-29 Family Suppresses the Invasion of HT1080 Human Fibrosarcoma Cells by Regulating Matrix Metalloproteinase 2 Expression. Chonnam Med. J..

[115] Liu P., Wilson M.J. (2012). miR-520c and miR-373 upregulate MMP9 expression by targeting mTOR and SIRT1, and activate the Ras/Raf/MEK/Erk signaling pathway and NF-κB factor in human fibrosarcoma cells. J. Cell. Physiol..

[116] Wong P., Hui A., Su J., Yue S., Haibe-Kains B., Gokgoz N., Xu W., Bruce J., Williams J., Catton C. (2015). Prognostic microRNAs modulate the RHO adhesion pathway: A potential therapeutic target in undifferentiated pleomorphic sarcomas. Oncotarget.

[117] Papp G., Krausz T., Stricker T.P., Szendrői M., Sápi Z. (2014). SMARCB1 expression in epithelioid sarcoma is regulated by miR-206, miR-381, and miR-671-5p on Both mRNA and protein levels. Genes Chromosomes Cancer.

[118] O’Hara A.J., Wang L., Dezube B.J., Harrington W.J.J., Damania B., Dittmer D.P. (2009). Tumor suppressor microRNAs are underrepresented in primary effusion lymphoma and Kaposi sarcoma. Blood.

[119] Wu Y.-H., Hu T.-F., Chen Y.-C., Tsai Y.-N., Tsai Y.-H., Cheng C.-C., Wang H.-W. (2011). The manipulation of miRNA-gene regulatory networks by KSHV induces endothelial cell motility. Blood.

[120] O’Hara A.J., Chugh P., Wang L., Netto E.M., Luz E., Harrington W.J., Dezube B.J., Damania B., Dittmer D.P. (2009). Pre-Micro RNA Signatures Delineate Stages of Endothelial Cell Transformation in Kaposi Sarcoma. PLoS Pathog..

[121] Greither T., Würl P., Grochola L., Bond G., Bache M., Kappler M., Lautenschläger C., Holzhausen H.-J., Wach S., Eckert A.W. (2012). Expression of microRNA 210 associates with poor survival and age of tumor onset of soft-tissue sarcoma patients. Int. J. Cancer.

[122] Bao X., Ren T., Huang Y., Sun K., Wang S., Liu K., Zheng B., Guo W. (2017). Knockdown of long non-coding RNA HOTAIR increases miR-454-3p by targeting Stat3 and Atg12 to inhibit chondrosarcoma growth. Cell Death Dis..

[123] Shao Y., Zhang Y., Hou Y., Tong H., Zhuang R., Ji Z., Wang B., Zhou Y., Lu W. (2017). A novel long noncoding RNA PILRLS promote proliferation through TCL1A by activing MDM2 in Retroperitoneal liposarcoma. Oncotarget.

[124] He R.-Q., Wei Q.-J., Tang R.-X., Chen W.-J., Yang X., Peng Z.-G., Hu X.-H., Ma J., Chen G. (2017). Prediction of clinical outcome and survival in soft-tissue sarcoma using a ten-lncRNA signature. Oncotarget.

[125] Barthel F.P., Wei W., Tang M., Martinez-Ledesma E., Hu X., Amin S.B., Akdemir K.C., Seth S., Song X., Wang Q. (2017). Systematic analysis of telomere length and somatic alterations in 31 cancer types. Nat. Genet..

[126] Koelsche C., Renner M., Hartmann W., Brandt R., Lehner B., Waldburger N., Alldinger I., Schmitt T., Egerer G., Penzel R. (2014). TERT promoter hotspot mutations are recurrent in myxoid liposarcomas but rare in other soft tissue sarcoma entities. J. Exp. Clin. Cancer Res..

[127] Dubbink H.J., Bakels H., Post E., Zwarthoff E.C., Verdijk R.M. (2014). TERT promoter mutations and BRAF mutations are rare in sporadic, and TERT promoter mutations are absent in NF1-related malignant peripheral nerve sheath tumors. J. Neuro Oncol..

[128] Castelo-Branco P., Leão R., Lipman T., Campbell B., Lee D., Price A., Zhang C., Heidari A., Stephens D., Boerno S. (2016). A cancer specific hypermethylation signature of the TERT promoter predicts biochemical relapse in prostate cancer: A retrospective cohort study. Oncotarget.

[129] Yoo J., Robinson R.A. (2000). Expression of telomerase activity and telomerase RNA in human soft tissue sarcomas. Arch. Pathol. Lab. Med..

[130] Bryan T.M., Englezou A., Dalla-Pozza L., Dunham M.A., Reddel R.R. (1997). Evidence for an alternative mechanism for maintaining telomere length in human tumors and tumor-derived cell lines. Nat. Med..

[131] Costa A., Daidone M.G., Daprai L., Villa R., Cantù S., Pilotti S., Mariani L., Gronchi A., Henson J.D., Reddel R.R. (2006). Telomere maintenance mechanisms in liposarcomas: Association with histologic subtypes and disease progression. Cancer Res..

[132] Henson J.D., Hannay J.A., McCarthy S.W., Royds J.A., Yeager T.R., Robinson R.A., Wharton S.B., Jellinek D.A., Arbuckle S.M., Yoo J. (2005). A robust assay for alternative lengthening of telomeres in tumors shows the significance of alternative lengthening of telomeres in sarcomas and astrocytomas. Clin. Cancer Res. Off. J. Am. Assoc. Cancer Res..

[133] Heaphy C.M., Subhawong A.P., Hong S.-M., Goggins M.G., Montgomery E.A., Gabrielson E., Netto G.J., Epstein J.I., Lotan T.L., Westra W.H. (2011). Prevalence of the alternative lengthening of telomeres telomere maintenance mechanism in human cancer subtypes. Am. J. Pathol..

[134] Liau J.-Y., Tsai J.-H., Yang C.-Y., Lee J.-C., Liang C.-W., Hsu H.-H., Jeng Y.-M. (2015). Alternative lengthening of telomeres phenotype in malignant vascular tumors is highly associated with loss of ATRX expression and is frequently observed in hepatic angiosarcomas. Hum. Pathol..

[135] Liau J.-Y., Tsai J.-H., Jeng Y.-M., Lee J.-C., Hsu H.-H., Yang C.-Y. (2015). Leiomyosarcoma with alternative lengthening of telomeres is associated with aggressive histologic features, loss of ATRX expression, and poor clinical outcome. Am. J. Surg. Pathol..

[136] Steele C.D., Tarabichi M., Oukrif D., Webster A.P., Ye H., Fittall M., Lombard P., Martincorena I., Tarpey P.S., Collord G. (2019). Undifferentiated Sarcomas Develop through Distinct Evolutionary Pathways. Cancer Cell.

[137] Miolo G., Di Gregorio E., Saorin A., Lombardi D., Scalone S., Buonadonna A., Steffan A., Corona G. (2020). Integration of serum metabolomics into clinical assessment to improve outcome prediction of metastatic soft tissue sarcoma patients treated with trabectedin. Cancers.

[138] Kowalczyk T., Ciborowski M., Kisluk J., Kretowski A., Barbas C. (2020). Mass spectrometry based proteomics and metabolomics in personalized oncology. Biochim. Biophys. Acta Mol. Basis Dis..

[139] Wishart D.S. (2019). Metabolomics for investigating physiological and pathophysiological processes. Physiol. Rev..

[140] Corona G., Rizzolio F., Giordano A., Toffoli G. (2012). Pharmaco-metabolomics: An emerging “omics” tool for the personalization of anticancer treatments and identification of new valuable therapeutic targets. J. Cell. Physiol..

[141] Wishart D.S., Feunang Y.D., Marcu A., Guo A.C., Liang K., Vázquez-Fresno R., Sajed T., Johnson D., Li C., Karu N. (2018). HMDB 4.0: The human metabolome database for 2018. Nucleic Acids Res..

[142] Ulrich E.L., Akutsu H., Doreleijers J.F., Harano Y., Ioannidis Y.E., Lin J., Livny M., Mading S., Maziuk D., Miller Z. (2008). BioMagResBank. Nucleic Acids Res..

[143] SDBS Spectral Database for Organic Compounds. https://sdbs.db.aist.go.jp/sdbs/cgi-bin/cre_index.cgi.

[144] Chenomx Chenomx NMR Suite 7.0 (Chenomx, Edmonton, Canada). https://www.chenomx.com/.

[145] BBIOREFCODE Metabolite Reference Database. https://www.bruker.com/en/products-and-solutions/mr/nmr-clinical-research-solutions/bbiorefcode.html.

[146] MetaboBase Bruker MetatoBase Personal Lybrary. https://bruker-labscape.store/products/bruker-metabobase-personal-library-3-0.

[147] Dona A.C., Kyriakides M., Scott F., Shephard E.A., Varshavi D., Veselkov K., Everett J.R. (2016). A guide to the identification of metabolites in NMR-based metabonomics/metabolomics experiments. Comput. Struct. Biotechnol. J..

[148] Huang X., Powers R., Tymiak A., Espina R., Roongta V. (2007). Introduction to NMR and Its Application in Metabolite Structure Determination. Drug Metabolism in Drug Design and Development.

[149] Bitchagno G.T.M., Fobofou Tanemossu S.A. (2019). Computational methods for NMR and MS for structure elucidation III: More advanced approaches. Phys. Sci. Rev..

[150] Boiteau R.M., Hoyt D.W., Nicora C.D., Kinmonth-Schultz H.A., Ward J.K., Bingol K. (2018). Structure elucidation of unknown metabolites in metabolomics by combined NMR and MS/MS prediction. Metabolites.

[151] Lou S., Balluff B., Cleven A.H.G., Bovée J.V.M.G., McDonnell L.A. (2017). Prognostic Metabolite Biomarkers for Soft Tissue Sarcomas Discovered by Mass Spectrometry Imaging. J. Am. Soc. Mass Spectrom..

[152] Schrimpe-Rutledge A.C., Codreanu S.G., Sherrod S.D., McLean J.A. (2016). Untargeted Metabolomics Strategies—Challenges and Emerging Directions. J. Am. Soc. Mass Spectrom..

[153] MetaboLights. https://www.ebi.ac.uk/metabolights/.

[154] Zhang W.T., Zhang Z.W., Guo Y.D., Wang L.S., Mao S.Y., Zhang J.F., Liu M.N., Yao X.D. (2018). Discovering biomarkers in bladder cancer by metabolomics. Biomark. Med..

[155] Corona G., Cannizzaro R., Miolo G., Caggiari L., De Zorzi M., Repetto O., Steffan A., De Re V. (2018). Use of metabolomics as a complementary omic approach to implement risk criteria for first-degree relatives of gastric cancer patients. Int. J. Mol. Sci..

[156] Vignoli A., Muraro E., Miolo G., Tenori L., Turano P., Di Gregorio E., Steffan A., Luchinat C., Corona G. (2020). Effect of estrogen receptor status on circulatory immune and metabolomics profiles of her2-positive breast cancer patients enrolled for neoadjuvant targeted chemotherapy. Cancers.

[157] Abooshahab R., Gholami M., Sanoie M., Azizi F., Hedayati M. (2019). Advances in metabolomics of thyroid cancer diagnosis and metabolic regulation. Endocrine.

[158] Roy J., Dibaeinia P., Fan T.M., Sinha S., Das A. (2019). Global analysis of osteosarcoma lipidomes reveal altered lipid profiles in metastatic versus nonmetastatic cells. J. Lipid Res..

[159] Kelly A.D., Breitkopf S.B., Yuan M., Goldsmith J., Spentzos D., Asara J.M. (2011). Metabolomic profiling from formalin-fixed, paraffin-embedded tumor tissue using targeted LC/MS/MS: Application in sarcoma. PLoS ONE.

[160] Braas D., Ahler E., Tam B., Nathanson D., Riedinger M., Benz M.R., Smith K.B., Eilber F.C., Witte O.N., Tap W.D. (2012). Metabolomics strategy reveals subpopulation of liposarcomas sensitive to gemcitabine treatment. Cancer Discov..

[161] Lee P., Malik D., Perkons N., Huangyang P., Khare S., Rhoades S., Gong Y.Y., Burrows M., Finan J.M., Nissim I. (2020). Targeting glutamine metabolism slows soft tissue sarcoma growth. Nat. Commun..

[162] Le A., Lane A.N., Hamaker M., Bose S., Gouw A., Barbi J., Tsukamoto T., Rojas C.J., Slusher B.S., Zhang H. (2012). Glucose-independent glutamine metabolism via TCA cycling for proliferation and survival in b cells. Cell Metab..

[163] Son J., Lyssiotis C.A., Ying H., Wang X., Hua S., Ligorio M., Perera R.M., Ferrone C.R., Mullarky E., Shyh-Chang N. (2013). Glutamine supports pancreatic cancer growth through a KRAS-regulated metabolic pathway. Nature.

[164] Birsoy K., Wang T., Chen W.W., Freinkman E., Abu-Remaileh M., Sabatini D.M. (2015). An Essential Role of the Mitochondrial Electron Transport Chain in Cell Proliferation Is to Enable Aspartate Synthesis. Cell.

[165] Sullivan L.B., Gui D.Y., Hosios A.M., Bush L.N., Freinkman E., Vander Heiden M.G. (2015). Supporting Aspartate Biosynthesis Is an Essential Function of Respiration in Proliferating Cells. Cell.

[166] Tardito S., Oudin A., Ahmed S.U., Fack F., Keunen O., Zheng L., Miletic H., Sakariassen P.Ø., Weinstock A., Wagner A. (2015). Glutamine synthetase activity fuels nucleotide biosynthesis and supports growth of glutamine-restricted glioblastoma. Nat. Cell Biol..

[167] Curis E., Crenn P., Cynober L. (2007). Citrulline and the gut. Curr. Opin. Clin. Nutr. Metab. Care.

[168] Miolo G., Basile D., Carretta A., Santeufemia D.A., Steffan A., Corona G. (2019). The metabolomic scent of cancer disease progression in soft tissue sarcoma: A case report. Int. J. Biol. Markers.

[169] Jia B., Wang W., Lin S., Shi L., Li Y., Gu Y., Gao F., Qin Y. (2020). The free amino acid profiles and metabolic biomarkers of predicting the chemotherapeutic response in advanced sarcoma patients. Clin. Transl. Oncol..

[170] Phillips M.M., Sheaff M.T., Szlosarek P.W. (2013). Targeting arginine-dependent cancers with arginine-degrading enzymes: Opportunities and challenges. Cancer Res. Treat..

[171] Zhang Z., Qiu Y., Hua Y., Wang Y., Chen T., Zhao A., Chi Y., Pan L., Hu S., Li J. (2010). Serum and urinary metabonomic study of human osteosarcoma. J. Proteome Res..

[172] López-Garrido L., Bañuelos-Hernández A.E., Pérez-Hernández E., Tecualt-Gómez R., Quiroz-Williams J., Ariza-Castolo A., Becerra-Martínez E., Pérez-Hernández N. (2020). Metabolic profiling of serum in patients with cartilage tumours using 1H-NMR spectroscopy: A pilot study. Magn. Reson. Chem..

[173] Merry E., Thway K., Jones R.L., Huang P.H. (2021). Predictive and prognostic transcriptomic biomarkers in soft tissue sarcomas. Jnp. Precis. Oncol..

[174] Min L., Shen J., Tu C., Hornicek F., Duan Z. (2016). The roles and implications of exosomes in sarcoma. Cancer Metastasis Rev..

[175] Grünewald T.G.P., Alonso M., Avnet S., Banito A., Burdach S., Cidre-Aranaz F., Di Pompo G., Distel M., Dorado-Garcia H., Garcia-Castro J. (2020). Sarcoma treatment in the era of molecular medicine. EMBO Mol. Med..

[176] Chinen L.T.D., Mello C.A.L., Abdallah E.A., Ocea L.M., Buim M.E., Breve N.M., Gasparini J.L.J., Fanelli M.F., Paterlini-Bréchot P. (2014). Isolation, detection, and immunomorphological characterization of circulating tumor cells (CTCs) from patients with different types of sarcoma using isolation by size of tumor cells: A window on sarcoma-cell invasion. OncoTargets Ther..

[177] Tellez-Gabriel M., Brown H.K., Young R., Heymann M.-F., Heymann D. (2016). The Challenges of Detecting Circulating Tumor Cells in Sarcoma. Front. Oncol..

[178] Martín-Broto J., Pousa A.L., Brohl A.S., Van Tine B.A., Powers B., Stacchiotti S., Blay J.-Y., Hu J.S., Oakley G.J., Wang H. (2021). Circulating Tumor Cells and Biomarker Modulation with Olaratumab Monotherapy Followed by Olaratumab plus Doxorubicin: Phase Ib Study in Patients with Soft-Tissue Sarcoma. Mol. Cancer Ther..

[179] Miranda-Castro R., Palchetti I., de-los-Santos-Álvarez N. (2020). The Translational Potential of Electrochemical DNA-Based Liquid Biopsy. Front. Chem..

[180] Sfragano P.S., Pillozzi S., Palchetti I. (2021). Electrochemical and PEC platforms for miRNA and other epigenetic markers of cancer diseases: Recent updates. Electrochem. Commun..

[181] Voccia D., Sosnowska M., Bettazzi F., Roscigno G., Fratini E., De Franciscis V., Condorelli G., Chitta R., D’Souza F., Kutner W. (2017). Direct determination of small RNAs using a biotinylated polythiophene impedimetric genosensor. Biosens. Bioelectron..

[182] Haddox C.L., Riedel R.F. (2020). Individualizing systemic therapy for advanced soft tissue sarcomas based on tumor histology and biology. Expert Rev. Anticancer Ther..

[183] Bertucci F., Finetti P., Monneur A., Perrot D., Chevreau C., Le Cesne A., Blay J.-Y., Mir O., Birnbaum D. (2019). PARP1 expression in soft tissue sarcomas is a poor-prognosis factor and a new potential therapeutic target. Mol. Oncol..

[184] Wilky B.A., Trucco M.M., Subhawong T.K., Florou V., Park W., Kwon D., Wieder E.D., Kolonias D., Rosenberg A.E., Kerr D.A. (2019). Axitinib plus pembrolizumab in patients with advanced sarcomas including alveolar soft-part sarcoma: A single-centre, single-arm, phase 2 trial. Lancet. Oncol..

[185] Van Tine B., Butler M., Araujo D., Johnson M., Clarke J., Liebner D., Odunsi K., Olszanski A., Basu S., Brophy F. (2019). 1670OADP-A2M4 (MAGE-A4) in patients with synovial sarcoma. Ann. Oncol..

[186] Grignani G., D’Ambrosio L., Pignochino Y., Palmerini E., Zucchetti M., Boccone P., Aliberti S., Stacchiotti S., Bertulli R., Piana R. (2018). Trabectedin and olaparib in patients with advanced and non-resectable bone and soft-tissue sarcomas (TOMAS): An open-label, phase 1b study from the Italian Sarcoma Group. Lancet. Oncol..

[187] Pignochino Y., Capozzi F., D’Ambrosio L., Dell’Aglio C., Basiricò M., Canta M., Lorenzato A., Vignolo Lutati F., Aliberti S., Palesandro E. (2017). PARP1 expression drives the synergistic antitumor activity of trabectedin and PARP1 inhibitors in sarcoma preclinical models. Mol. Cancer.

[188] Stacchiotti S., Schoffski P., Jones R., Agulnik M., Villalobos V.M., Jahan T.M., Chen T.W.-W., Italiano A., Demetri G.D., Cote G.M. (2019). Safety and efficacy of tazemetostat, a first-in-class EZH2 inhibitor, in patients (pts) with epithelioid sarcoma (ES) (NCT02601950). J. Clin. Oncol..

[189] Wagner A.J., Ravi V., Ganjoo K.N., Van Tine B.A., Riedel R.F., Chugh R., Cranmer L.D., Gordon E.M., Hornick J.L., Kwiatkowski D.J. (2019). ABI-009 (nab-sirolimus) in advanced malignant perivascular epithelioid cell tumors (PEComa): Preliminary efficacy, safety, and mutational status from AMPECT, an open label phase II registration trial. J. Clin. Oncol..

[190] Dickson M.A., Koff A., D’Angelo S.P., Gounder M.M., Keohan M.L., Kelly C.M., Chi P., Antonescu C.R., Landa J., Qin L.-X. (2019). Phase 2 study of the CDK4 inhibitor abemaciclib in dedifferentiated liposarcoma. J. Clin. Oncol..

[191] Groisberg R., Hong D.S., Holla V., Janku F., Piha-Paul S., Ravi V., Benjamin R., Kumar Patel S., Somaiah N., Conley A. (2017). Clinical genomic profiling to identify actionable alterations for investigational therapies in patients with diverse sarcomas. Oncotarget.

[192] Lucchesi C., Khalifa E., Laizet Y., Soubeyran I., Mathoulin-Pelissier S., Chomienne C., Italiano A. (2018). Targetable Alterations in Adult Patients with Soft-Tissue Sarcomas: Insights for Personalized Therapy. JAMA Oncol..

[193] Drilon A., Laetsch T.W., Kummar S., DuBois S.G., Lassen U.N., Demetri G.D., Nathenson M., Doebele R.C., Farago A.F., Pappo A.S. (2018). Efficacy of Larotrectinib in TRK Fusion-Positive Cancers in Adults and Children. N. Engl. J. Med..

